# Coffee Pulp, a By-Product of Coffee Production, Modulates Gut Microbiota and Improves Metabolic Syndrome in High-Carbohydrate, High-Fat Diet-Fed Rats

**DOI:** 10.3390/pathogens10111369

**Published:** 2021-10-22

**Authors:** Nikhil S. Bhandarkar, Peter Mouatt, Marwan E. Majzoub, Torsten Thomas, Lindsay Brown, Sunil K. Panchal

**Affiliations:** 1 Functional Foods Research Group, University of Southern Queensland, Toowoomba, QLD 4350, Australia; bhandark@post.bgu.ac.il (N.S.B.); lindsay.brown@griffith.edu.au (L.B.); 2 Southern Cross Plant Science, Southern Cross University, Lismore, NSW 2480, Australia; Peter.Mouatt@scu.edu.au; 3 Centre for Marine Science and Innovation, School of Biological, Earth and Environmental Sciences, University of New South Wales, Sydney, NSW 2052, Australia; m.majzoub@unsw.edu.au (M.E.M.); t.thomas@unsw.edu.au (T.T.)

**Keywords:** coffee pulp, chlorogenic acid, obesity, metabolic syndrome, high-carbohydrate, high-fat, gut microbiota

## Abstract

Waste from food production can be re-purposed as raw material for usable products to decrease industrial waste. Coffee pulp is 29% of the dry weight of coffee cherries and contains caffeine, chlorogenic acid, trigonelline, diterpenes and fibre. We investigated the attenuation of signs of metabolic syndrome induced by high-carbohydrate, high-fat diet in rats by dietary supplementation with 5% freeze-dried coffee pulp for the final 8 weeks of a 16-week protocol. Coffee pulp decreased body weight, feed efficiency and abdominal fat; normalised systolic blood pressure, left ventricular diastolic stiffness, and plasma concentrations of triglycerides and non-esterified fatty acids; and improved glucose tolerance in rats fed high-carbohydrate, high-fat diet. Further, the gut microbiota was modulated with high-carbohydrate, high-fat diet and coffee pulp supplementation and 14 physiological parameters were correlated with the changes in bacterial community structures. This study suggested that coffee pulp, as a waste from the coffee industry, is useful as a functional food for improving obesity-associated metabolic, cardiovascular and liver structure and function, and gut microbiota.

## 1. Introduction

Coffee is an important agricultural commodity, with an average price of 141 US cents/lb in June 2021 [1]. Total exports of coffee worldwide accounted for 7.7 million tonnes from June 2020 to May 2021, with an estimated production of over 10 million tonnes [1]. Most coffee is grown in tropical developing countries, with Brazil, Vietnam and Colombia as the three major producers [2]. Coffee production is a multi-step process including six low-technology steps of pulping, fermentation, drying, hulling, roasting, then grinding [3,4]. However, these processes generate over 20 million tonnes of liquid and solid waste each year, as the yield of green coffee is about 150–200 kg/tonne of coffee cherry [5], causing environmental pollution in rural areas with limited opportunities to remediate this waste [6]. Value-added products can be recovered from coffee waste [7], with two general concepts as the production of biogas and the use of oxygen-driven biological methods, such as composting to improve soil fertility [8]. Coffee waste includes coffee pulp, which represents 29% of the weight of the whole berry [9]. This pulp has long been considered as a substitute but usually inferior food for monogastric animals, poultry, and ruminants [10] as well as readily available fertiliser for coffee crops [11].

Studies on potential health benefits of coffee have concentrated on roasted coffee as a beverage rather than intermediate products from the preparation, such as coffee pulp from coffee cherry. An umbrella analysis of 201 meta-analyses of observational research and 17 meta-analyses of interventional research with roasted coffee indicated the largest relative risk reductions at intakes of three to four cups daily, including in all-cause mortality, cardiovascular disease and mortality, and incident cancer [12]. Our previous study with another coffee waste, spent coffee grounds, has shown modulation of gut microbiota that could contribute to the beneficial effects in improving metabolic syndrome [13]. The few studies on coffee pulp indicate that it may improve health as an antioxidant [14]; further, it prevented release of interleukin-8 from human epithelial gastric cells as a possible anti-inflammatory response [15], lowered cholesterol both in vitro and in vivo [16], and improved liver steatosis [17]. This information suggests that coffee pulp could be useful in the treatment of metabolic syndrome, a chronic low-grade inflammatory state [18], as a constellation of hypertension, central obesity, dyslipidaemia, impaired glucose tolerance and insulin resistance that further increases the risk of developing type 2 diabetes, cardiovascular diseases and non-alcoholic fatty liver disease [19,20]. Improvements in metabolic syndrome could then provide additional value to coffee growers from a current waste product [21].

As in our study on spent coffee grounds [13], the aim of this study was to determine whether coffee pulp can improve components of metabolic syndrome when given as an 8-week dietary intervention in rats fed a high-carbohydrate, high-fat diet for 16 weeks. We measured metabolic parameters related to obesity and glucose tolerance, cardiovascular function in vivo by measurement of systolic blood pressure and ex vivo in isolated Langendorff heart preparations together with histology, and liver structure and function by histology and plasma biochemistry. Further, we characterised the changes in the composition of the gut microbiota, as functional foods may reverse obesity-induced microbiota changes [22]. Our hypothesis was that addition of coffee pulp to the diet will reverse the metabolic, cardiovascular, liver and gut microbiota changes in rats with diet-induced metabolic syndrome.

## 2. Results

### 2.1. Intake of Coffee Pulp Components

Freeze-dried powder of coffee pulp contained 0.58% (*w*/*w*) caffeine, 0.34% (*w*/*w*) trigonelline, 2.51% (*w*/*w*) diterpenes (cafestol, kahweol and their esters), 0.10% (*w*/*w*) chlorogenic acid and 0.19% (*w*/*w*) phenolic acids (Figures S1–S4). The caffeine, chlorogenic acid, phenolic acids, trigonelline and diterpenes doses were higher in corn starch diet-fed rats treated with coffee pulp (CCP) than in high-carbohydrate, high-fat diet-fed rats (HCP) due to higher intake of food by CCP rats (Table 1).

### 2.2. Dietary Intake, Body Composition, and Plasma Biochemistry

Body weight was similar between corn starch diet-fed rats (C) and CCP rats. High-carbohydrate, high-fat diet-fed rats (H) increased body weight compared to C rats, while coffee pulp treatment decreased body weight in HCP rats compared to H rats (Table 2). Food intake was lower in H rats compared to C rats and coffee pulp did not change food intake in both CCP and HCP rats (Table 2). Water intake was similar between C, H and HCP rats while CCP rats had higher water intake. Energy intake was higher in H rats compared to C rats. Coffee pulp did not change energy intake in CCP rats compared to C rats, whereas it was higher in HCP rats compared to H rats (Table 2). Coffee pulp did not change feed efficiency, decreased abdominal circumference and increased body mass index in CCP rats compared to C rats. Feed efficiency, body mass index and abdominal circumference were higher in H rats than in C rats. Coffee pulp reduced these parameters in HCP rats compared to H rats (Table 2). Heat produced and respiratory exchange ratio were unchanged by coffee pulp in both CCP and HCP rats. Basal blood glucose concentrations were higher in H rats compared to C rats. Coffee pulp reduced basal blood glucose concentrations in both CCP and HCP rats compared to C and H rats, respectively. Area under the curve was higher in H rats compared to C rats, while it was decreased in both CCP and HCP rats compared to C and H rats, respectively (Table 2). Whole-body lean mass was similar between C and H rats and coffee pulp only increased it in HCP rats. Whole-body fat mass was higher in H rats than in C rats but decreased with coffee pulp in HCP rats compared to H rats (Table 2). Retroperitoneal, epididymal, omental and total abdominal fat pads were unchanged in CCP rats compared to C rats but were higher in H rats than in C rats. Coffee pulp reduced total abdominal fat in HCP rats through reduction in omental and epididymal fat pads (Table 2). Plasma total cholesterol concentrations were lower in CCP rats compared to C, H and HCP rats, while coffee pulp reduced plasma concentrations of triglycerides and non-esterified fatty acids in HCP rats compared to H rats (Table 2). Intestinal transit rate was decreased by coffee pulp in HCP rats compared to H rats (Table 2).

### 2.3. Cardiovascular Structure and Function

**Figure 1:**
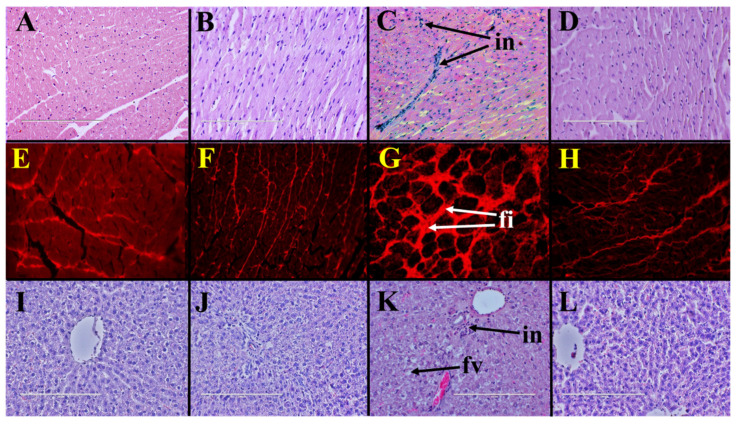
Coffee pulp responses on heart and liver structure. Haematoxylin and eosin staining (×20) showing infiltration of inflammatory cells (“in”) and picrosirius red staining (×20) showing fibrosis (“fi”) in left ventricles from high-carbohydrate, high-fat diet-fed rats (**C**,**G**) compared to corn starch diet-fed rats (**A**,**E**); corn starch diet-fed rats supplemented with coffee pulp (**B**,**F**); and high-carbohydrate, high-fat diet-fed rats supplemented with coffee pulp (**D**,**H**). Haematoxylin and eosin staining (×20) showing enlarged fat vacuoles (“fv”) in livers from high-carbohydrate, high-fat diet-fed rats (**K**) compared to corn starch diet-fed rats (**I**), corn starch diet-fed rats supplemented with coffee pulp (**J**) and high-carbohydrate, high-fat diet-fed rats supplemented with coffee pulp (**L**).

Histopathological analysis of left ventricle indicated increased infiltration of inflammatory cells in H rats (Figure 1C) compared to C rats (Figure 1A). Coffee pulp reduced infiltration of inflammatory cells into the left ventricle of HCP rats (Figure 1D) compared to H rats. Picrosirius red staining of left ventricle showed higher collagen deposition in H rats (Figure 1G) compared to C rats (Figure 1E). Coffee pulp reduced collagen deposition in the left ventricle of HCP rats (Figure 1H) compared to H rats. Systolic blood pressure and left ventricular diastolic stiffness were higher in H rats than in C rats and coffee pulp reduced these parameters in HCP rats compared to H rats (Table 2). Left ventricular + septum and right ventricular wet weights were unchanged by coffee pulp treatment (Table 2).

### 2.4. Liver Structure and Function

Liver wet weight was higher in H rats than in C rats and coffee pulp decreased the liver wet weight of HCP rats compared to H rats (Table 2). Liver wet weight was unchanged by coffee pulp treatment between C and CCP rats. Increased inflammatory cell infiltration and fat deposition were observed in livers from H rats (Figure 1K) compared to C rats (Figure 1I). Coffee pulp reduced infiltration of inflammatory cells and fat deposition in livers of HCP rats (Figure 1L) compared to H rats. Plasma alanine transaminase activity was unchanged between the groups. Plasma aspartate transaminase activity was unchanged between H and C rats and increased in HCP rats compared to H rats (Table 2).

### 2.5. Gut Microbiota

A 16S rRNA gene-based analysis was used to assess the bacterial communities (Figure S5). There were no differences in Shannon’s diversity index for the four different feeding regimes. Higher richness was observed for the C samples, and this difference was more pronounced for C compared to H samples (*p* = 0.0083 to 0.0034 for H and HCP, respectively) (Figure S6).

**Figure 2:**
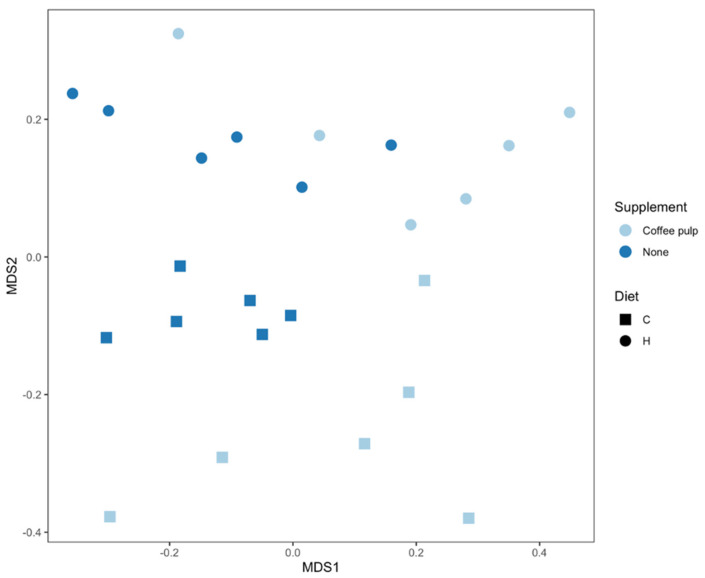
nMDS plot of bacterial community structure of faecal samples. C, corn starch diet-fed rats; H, high-carbohydrate, high-fat diet-fed rats.

An effect of diet and treatment was observed on the overall bacterial community structure based on Bray–Curtis dissimilarity (Figure 2, Table S1; PERMANOVA, both *p* = 0.0001), as well as the interaction of diet and treatment (PERMANOVA, *p* = 0.017). There were differences between C and H samples (*p* = 0.012) indicating an effect of basal diet on the bacterial community structure. There was also an effect of the addition of coffee pulp with the C diet (*p* = 0.024; Figure 2, Table S1). Bacterial communities in the CCP treatment were also more variable between replicates compared to the C rats (Figure 2, Table S1; PERMDISP, *p* = 0.03).

**Figure 3:**
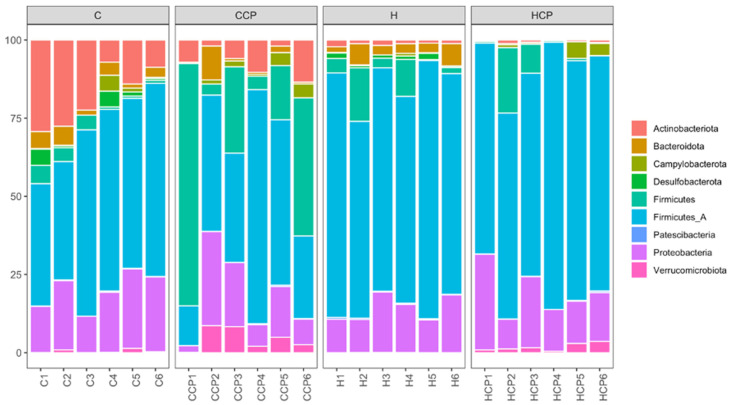
Taxonomic profiles of bacterial communities shown at the phylum level of all faecal samples.

Bacteria belonging to the phyla Actinobacteriota, Bacteroidota, Campylobacterota, Desulfobacterota, Firmicutes A, Firmicutes, Patescibacteria, Proteobacteria and Verrucomicrobiota were found in the faecal samples for the different treatment groups (Figure 3). Analysis of the bacterial community structure at the class level showed that Actinomycetia, Bacilli, Bacteroidia, Campylobacteria, Clostridia, Coriobacteriia, Desulfovibrionia, Gammaproteobacteria, Saccharimonadia and Verrucomicrobiae were detected in the faecal samples for the different treatment groups (Figure S7). Similarly, bacteria belonging to the families *Akkermansiaceae* (class Verrucomicrobiae), *Arcobacteraceae* (class Campylobacteria), *Bifidobacteriaceae* (class Actinobacteria), *Clostridiaceae* (class Clostridia), *Desulfovibrionaceae* (class Deltaproteobacteria), *Enterobacteriaceae* (class Gammaproteobacteria), *Lachnospiraceae* (class Clostridia), *Lactobacillaceae* (class Bacilli), *Muribaculaceae* (class Bacteroidia), *Oscillospiraceae* (class Clostridia), *Peptostreptococcaceae* (class Clostridia), *Ruminococcaceae* (class Clostridia) and *Vibrionaceae* (class Gammaproteobacteria) were detected in all faecal samples (Figure S8).

Comparison between C and H (Table S2) showed that diet had a stronger effect than supplementation on the bacterial community structure at the level of zero-radius operational taxonomic unit (zOTU) by affecting the relative read abundances of 461 zOTUs (7.17% of total zOTUs) belonging mostly to the phylum Actinobacteriota. Bacteria belonging to the phylum Actinobacteriota (families: *Bifidobacteriaceae*, *Atopobiaceae* and *Eggerthellaceae*; species: *Bifidobacterium criceti*, *Bifidobacterium globosum*, *Olsenella phocaeensis* and *Adlercreutzia equolifaciens*), Bacteroidota (family: *Muribaculaceae*; species: *Muribaculum sp002492595*), Firmicutes (family: *Erysipelotrichaceae*; species: *Allobaculum stercoricanis* and *Faecalibaculum rodentium*), Firmicutes A (families: *Clostridiaceae*, *Lachnospiraceae* and *UBA1381*; species: *Clostridium saudiense*, *CAG-127 sp900319515* and *CAG-41 sp900066215*) and Proteobacteria (families: *Enterobacteriaceae* and *Vibrionaceae*; species: *Cronobacter sakazakii* and *Vibrio parahaemolyticus*) were enriched in C rats compared to H rats, while bacteria belonging to the phylum Firmicutes A (families: *Lachnospiraceae* and *Peptostreptococcaceae*; species: *Eubacterium F sp003491505* and *Romboutsia ilealis*) were enriched in H rats compared to C rats.

Comparison between C and CCP (Table S3) showed that the relative abundances of 119 zOTUs (1.85% of total zOTUs) belonging mostly to the phylum Proteobacteria were affected by coffee pulp supplementation in CCP rats. Bacteria belonging to the phylum Actinobacteriota (families: *Bifidobacteriaceae* and *Eggerthellaceae*; species: *Bifidobacterium criceti*, *Bifidobacterium globosum* and *CAG-1427 sp000435675*), Bacteroidota (family: *Muribaculaceae*; species: *Muribaculum intestinale*, *Muribaculum sp001701195*, *Muribaculum sp002492595*, *UBA7173 sp004102805* and *Vibrio parahaemolyticus*), Firmicutes A (families: *Lachnospiraceae*, *UBA1381* and *Oscillospiraceae*; species: *1XD8-76 sp003611955*, *Roseburia intestinalis*, *CAG-41 sp900066215* and *CAG-83 sp000431575*) and Proteobacteria (families: *Enterobacteriaceae* and *Vibrionaceae*; species: *Cronobacter sakazakii*, *Salmonella enterica* and *Vibrio parahaemolyticus*) were enriched in C rats compared to CCP rats.

At the zOTU level and comparing H and HCP samples (Table S4), bacteria belonging to the phylum Actinobacteriota (family: *Eggerthellaceae*; species: *CAG-1427 sp000435675*), Firmicutes A (families: *Clostridiaceae* and *Lachnospiraceae*; species: *Clostridium saudiense*), Proteobacteria (family: *Enterobacteriaceae*; species: *Cronobacter malonaticus* and *Cronobacter sakazakii*) and Verrucomicrobiota (family: *Akkermansiaceae*; species: *Akkermansia muciniphila*) were enriched in HCP rats compared to H rats, while bacteria belonging to the phylum Bacteroidota (family: *Muribaculaceae*; species: *Muribaculum sp001701195*, *Muribaculum sp002492595*, *Vibrio parahaemolyticus* and *UBA7173 sp004102805*), Desulfobacterota (family: *Desulfovibrionaceae*; species: *Desulfovibrio sp003860215* and *Desulfovibrio sp900547595*) and Firmicutes A (family: *Lachnospiraceae*; species: *Acetatifactor sp900066565* and *Kineothrix alysoides*) were enriched in H rats compared to HCP rats.

Combined analysis of bacterial community structure and physiological parameters was performed. Mantel test revealed that, overall, the bacterial community structure and the physiological data are correlated (Mantel statistic r = 0.3498; *p*-value = 0.0001). Table 3 and Figure S9 show that individual physiological parameters contribute to the differences in bacterial community structure between treatments (function envfit – vegan R package). Food intake, water intake, feed efficiency, systolic blood pressure (week 16), liver wet weight and alanine transaminase activity were found to have the highest correlation with bacterial community structure (Figure S9).

## 3. Discussion

In this study, coffee pulp reduced body weight and central obesity and improved glucose tolerance along with improved heart and liver structure in diet-induced obese rats. Further, coffee pulp increased lean mass and decreased plasma lipids in obese rats. Diet and coffee pulp supplementation were also associated with changes in bacterial community structures of gut microbiota. These bacterial community structure changes were correlated with changes in 14 physiological parameters. The rat model of diet-induced metabolic syndrome used in this study has been validated for the signs of metabolic syndrome in humans, including abdominal obesity, impaired glucose tolerance, dyslipidaemia, elevated blood pressure, cardiovascular remodelling and fatty liver [23]. These responses of coffee pulp support intervention with this product as a functional food to provide benefits in the complex condition of human metabolic syndrome.

During coffee processing, large amounts of waste products, such as pulp, are generated which creates a serious environmental issue in coffee-growing areas [24,25]. Therefore, alternative applications for coffee pulp are extremely important. Coffee pulp has been used for production of cascara, a refreshing beverage containing 226 mg/L caffeine [26]. It has also been used for bioethanol production, as it contained polysaccharides, including arabinose, galactose, glucose, xylose and mannose, at concentrations of 5.8, 5.2, 20.2, 4.2 and 4.7% in dry mass, respectively [27]. Coffee pulp contained proteins 12%, fibre 41%, caffeine 1.8% and 1% polyphenols [14]. Recently, coffee pulp has been used in the production of fermented, alcohol-based beverages [28]. Moreover, use of coffee pulp for topical formulations in the pharmaceutical and cosmetic industry has been reviewed, suggesting their usefulness due to their low cost, sustainability, safety and effectiveness [29]. There are many studies confirming in vitro antioxidant activities of coffee pulp [14,24,30,31,32], but there are very few studies of coffee pulp determining in vivo benefits. Thus, this study is an important step towards the identification of the health benefits of coffee pulp.

The most likely bioactive components in coffee pulp are caffeine, chlorogenic acid, trigonelline, diterpenoids and fibre. HPLC analysis of coffee pulp showed phenolic compounds, such as chlorogenic acid (5-caffeoyl-quinic acid, 42.2%), epicatechin (21.6%), isochlorogenic acid I (5.7%), isochlorogenic acid II (19.3%), isochlorogenic acid III (4.4%), catechin (2.2%), rutin (2.1%), protocatechuic acid (1.6%) and ferulic acid (1.0%) [33]. Hence, the use of coffee pulp may be considered as a potential approach for management of metabolic complications associated with obesity, as there are many reports of health benefits from these individual components of coffee pulp [34]. However, there is insufficient evidence for the health effects of their combination as coffee pulp for use as a dietary supplement.

Meta-analysis of coffee consumption has reported the inverse relationship with components of metabolic syndrome, including abdominal obesity, hypertension, insulin resistance, impaired glucose tolerance, dyslipidaemia and liver complications [35,36]. Treatment with coffee extract attenuated high-fat diet-induced metabolic disorders and decreased body weight, adipose tissue, and plasma concentrations of glucose, free fatty acid, cholesterol and insulin in mice [37]. In high-carbohydrate, high-fat diet-fed rats, coffee extract [38], caffeine [39], chlorogenic acid [40], green coffee [41] and spent coffee grounds [13] attenuated metabolic syndrome in these obese hypertensive rats. This study was conducted based on the concept that coffee pulp is a rich source of some of the same bioactive compounds. While coffee pulp has similar active ingredients as other coffee products, including caffeine, chlorogenic acids, phenolic acids and diterpenes, the proportion of bioactive compounds is different from coffee extract [38], green coffee [41] and spent coffee grounds [13].

Chlorogenic acid treatment (5 mg/kg/day) for three weeks in obese rats improved glucose tolerance, decreased plasma and liver lipid profile and recovered mineral pool distribution [42]. Chlorogenic acid (5 mg/kg/day) for 45 days in rats reduced blood lipids including cholesterol, free fatty acids, triglycerides, phospholipids, LDL-cholesterol, VLDL-cholesterol, and increased concentrations of HDL-cholesterol and decreased HMG-CoA reductase activity [43]. In our previous study, we showed that a higher dose of chlorogenic acid (~100 mg/kg/day) was required to attenuate inflammation as well as cardiovascular, liver and metabolic abnormalities induced by the high-carbohydrate, high-fat diet [40]. In comparison, this study treated rats with a low dose of chlorogenic acid (~3 mg/kg/day), which means chlorogenic acid is unlikely to be the major bioactive component of coffee pulp. Caffeine treatment (~28 mg/kg/day) in obese rats for eight weeks improved structure and function of heart and liver, with a reduction in obesity except for dyslipidaemia [39]. Rats in this study received ~17 mg/kg/day of caffeine, which means it may contribute to the effects of coffee pulp. Caffeine antagonised A_1_-adenosine receptors in the hypothalamus to suppress appetite and promote energy use that reduced diet-induced obesity in mice [44].

Coffee pulp used in this study also contained trigonelline, the second most abundant alkaloid in green coffee beans. Trigonelline, as an antioxidant, attenuated endoplasmic reticulum-associated stress and oxidative stress-triggered damage in pancreas and adipocytes [45]. Trigonelline (40 mg/kg/day) treatment in the diet for eight weeks reduced serum activity of aspartate transaminase and aspartate transaminase and serum concentrations of total cholesterol and LDL-cholesterol and decreased non-alcoholic fatty liver diseases in rats fed with a high-fat diet [46]. Trigonelline reduced lipid accumulation by restricting adipocyte differentiation by the PPARγ cascade, along with inhibition of adipocyte differentiation through downregulation of fatty acid synthase and GLUT-4 transporter in muscles and adipocytes of mice [47]. These results suggest that trigonelline at a dose of 10 mg/kg/day, as in the current study, has the potential to attenuate metabolic syndrome.

Coffee contains diterpenes cafestol and kahweol. These diterpenes have been shown to be responsible for increases in human plasma triglycerides and low-density lipoprotein [48,49]. However, other studies have shown the opposite responses for coffee diterpenes [50]. As coffee contains very small amounts of these diterpenes, these responses are difficult to evaluate in studies with coffee, but these responses need to be confirmed in human studies with similar doses as received from coffee. Further, kahweol showed anti-inflammatory effect in carrageenan-induced acute air pouch inflammation model in rats [51]. Cafestol stimulated clonal rat insulinoma cell line (INS-1E) to secrete insulin and increase glucose uptake in muscle cells [52]. Cafestol treatment for 10 weeks reduced fasting blood glucose and fasting glucagon, promoted insulin secretion and increased insulin sensitivity in KKAy mice [53]. Kahweol reduced lipid accumulation in 3T3-L1 adipocytes and inhibited expression of genes involved in lipid accumulation [54,55]. Considering higher concentrations of diterpenes in coffee pulp, it is plausible that some of the beneficial responses observed in this study may be contributed by these compounds.

Apart from these above-mentioned compounds, coffee pulp also contained fibre [56]. Fibre reduced obesity-associated health disorders through modulation of gut microbiota [57] and reduced body fat [58]. Our study with coffee pulp intervention reduced plasma concentrations of triglycerides and non-esterified fatty acids along with the reduction in body fat mass. These changes may be associated with changes in gut microbiota that modulate the metabolic changes observed in obesity. Crude fibre accounted for approximately 33.6% [59] and 41% [14] of coffee pulp, and fibre has prebiotic effects on gut microbiota, leading to decreased food intake, weight gain and adiposity, increased circulating satiety hormones GLP-1 and PYY, and colonic fermentation [60]. Colonic fermentation of fibre produces butyrate, acetate and propionate, which are ligands for free fatty acid receptors [61]. Activation of free fatty acid receptors promotes expression and secretion of enteroendocrine hormones, such as glucagon-like-peptide 1 or peptide YY, which are responsible for satiety [62]. Thus, it can be expected that fibre contributes towards the benefits associated with coffee pulp intervention.

It is evident that gut microbiota plays an important role in health and disease development [63]. The changes introduced by high-carbohydrate, high-fat diet may be linked to lower availability of dietary fibre in the diet [64]. These changes were related to lower abundance of *Bifidobacterium criceti, Bifidobacterium globosum, Olsenella phocaeensis*, *Adlercreutzia equolifaciens*, *Muribaculum sp002492595*, *Allobaculum stercoricanis*, *Faecalibaculum rodentium*, *Clostridium saudiense, CAG-127 sp900319515*, *CAG-41 sp900066215* and *Cronobacter sakazakii*, and higher abundance of *Eubacterium F sp003491505* and *Romboutsia ilealis*. Further, coffee pulp intervention enriched the abundance of *Clostridium saudiense*, *Akkermansia muciniphila*, *Cronobacter malonaticus* and *Cronobacter sakazakii*. Some of these genera were shown to be involved in the development and treatment of obesity [65,66,67,68,69]. These changes in the microbiota were correlated with many metabolic changes, including changes in epididymal fat, omental fat, retroperitoneal fat, total abdominal fat, systolic blood pressure, plasma non-esterified fatty acids and body weight gain. This may suggest that coffee pulp can provide components that have their impact by improving gut microbiota composition.

Coffee pulp has great potential to be used in the development of functional foods for humans and animals. These functional food development opportunities may include cookies, muffins, breakfast cereal and coffee pulp flour for mixing with regular bread flour. The freeze-drying process can extend the storage time of this waste material that is currently discarded or composted. Products with coffee pulp have not shown any impact on taste perception [70,71,72,73].

## 4. Materials and Methods

### 4.1. Preparation of Coffee Pulp Powder

Coffee pulp sample (*Coffea arabica*) was collected from Mountain Top Coffee, Nimbin, NSW, Australia, in December 2016. The sample was freeze-dried at School of Agriculture and Food Sciences, The University of Queensland, Gatton, QLD, Australia, and stored at 4 °C until further processing and use as a food supplement.

### 4.2. Characterisation of Coffee Pulp Powder

Extracts of freeze-dried coffee pulp powder were prepared in 3:2 ethanol:water mixture. Briefly, 1 g of powder was dissolved in 50 mL of ethanol:water mixture, sonicated for 15 min and an aliquot of the supernatant was taken for analysis by HPLC using an Agilent 1200 series system coupled with a mass spectrometer for further peak confirmation or identification as required. The HPLC system consisted of a diode array detector (G4212B), binary pump (G4220A), an autosampler (G4226A), a vacuum degasser and a column oven with an MSD (G1946D) detector also present. The chromatography was performed on a Phenomenex luna C18 (2) HPLC column (100 × 4.6 mm) using a gradient method of water and acetonitrile with 0.005% trifluoroacetic acid over 28 min. The optimal solvent gradient for separation of target constituents started with 10% acetonitrile, which was increased as a gradient to 30% acetonitrile over 10 min, then to 95% acetonitrile over 8 min at a flow rate of 0.75 mL/minute and an injection volume of 5 µL. Calibration standards of trigonelline, caffeine, diterpenes [74,75] and chlorogenic acid were prepared in 3:2 ethanol:water at concentrations from 0.01 to 1 mg/mL, 0.005 to 0.5 mg/mL and 0.004 to 1 mg/mL for each of these standards, respectively. Specific detection and calibration curves for each compound were performed at 254 nm, 280 nm, 225 and 330 nm, respectively. Quantification was performed using the Chemstation Software based on reference standards, peak area and sample dilution at specific wavelengths for each compound.

### 4.3. Rats, Diets and Treatments

Male Wistar rats (8–9 weeks old, 340 ± 2 g, *n* = 48) were obtained from Animal Resource Centre, Perth, Australia. Rats were divided into 4 groups for the 16-week feeding protocol: corn starch diet-fed rats (C; *n* = 12); corn starch diet-fed rats supplemented with coffee pulp powder (CCP, *n* = 12; 5% in food for the final 8 weeks); high-carbohydrate, high-fat diet-fed rats (H; *n* = 12); and high-carbohydrate, high-fat diet-fed rats supplemented with coffee pulp powder (HCP, *n* = 12; 5% in food for the final 8 weeks). Corn starch diet contained 570 g corn starch; 155 g powdered rat food; 25 g Hubbel, Mendel & Wakeman salt mixture; and 250 g water per kilogram of diet. High-carbohydrate, high-fat diet contained 175 g fructose; 395 g sweetened condensed milk; 200 g beef tallow; 155 g powdered rat food; 25 g Hubbel, Mendel & Wakeman salt mixture and 50 g water per kilogram of food [23]. Drinking water with 25% (*w*/*v*) fructose was provided to H and HCP groups. C and CCP groups were given normal drinking water. The rats were individually housed under temperature-controlled, 12-h light/dark conditions and given free access to food and water [23].

### 4.4. Physiological Parameters in Live Rats

Body weight and food and water intakes were measured daily. Abdominal circumference and body length (nose to anus—for calculation of body mass index) were measured using a standard measuring tape under light anaesthesia with Zoletil (tiletamine 10 mg/kg, zolazepam 10 mg/kg, intraperitoneal). Body mass index was calculated as body weight (in grams)/(body length (in cm))^2^. Feed efficiency was calculated as (mean body weight gain (in grams)/daily energy intake (in kJ)) [23].

Systolic blood pressure was measured under light anaesthesia with Zoletil (tiletamine 10 mg/kg, zolazepam 10 mg/kg, intraperitoneally), using an MLT1010 Piezo-Electric Pulse Transducer and inflatable tail-cuff connected to an MLT844 Physiological Pressure Transducer and PowerLab data acquisition unit [23].

Dual-energy X-ray absorptiometric measurements were carried out at the end of the protocol with a Norland XR46 DXA instrument (Norland Corp., Fort Atkinson, WI, USA). These scans were evaluated using the manufacturer’s suggested software for use in laboratory animals (Small Subject Analysis Software, version 2.5.3/1.3.1; Norland Corp.) [23].

Oral glucose tolerance tests were performed after determining overnight fasting blood glucose concentrations in tail vein blood using Medisense Precision Q.I.D. glucose meters. For overnight fasting, rats were deprived of food for 12 h. Fructose-supplemented drinking water for H and HCP rats was replaced with normal drinking water for the overnight food-deprivation period. Basal blood glucose concentrations were measured followed by administration of glucose load 2 g/kg body weight as 40% glucose solution via oral gavage. Blood glucose concentrations were then measured 30, 60, 90 and 120 min after oral glucose administration [23].

Indirect calorimetry was applied to determine oxygen consumption and carbon dioxide production using a 4-chamber Oxy-Max system (Columbus Instruments, Columbus, OH, USA) with one rat per chamber. Rats were given free access to food and water during the measurements. Oxygen consumption (V_O2_) and carbon dioxide production (V_CO2_) were measured individually from each chamber. The respiratory exchange ratio was calculated by Oxy-Max software (v. 4.86). Energy expenditure was determined by assessment of the exchange of oxygen for carbon dioxide that occurs during the metabolic processing of food [76].

### 4.5. Measurements after Euthanasia

Rats were euthanised with Lethabarb (pentobarbitone sodium, 100 mg/kg, intraperitoneally). After induction of deep anaesthesia, heparin (200 IU) was injected through the right femoral vein. The abdomen was then opened and blood (~5 mL) was withdrawn from the abdominal aorta for collection into heparinised tubes. Blood was centrifuged at 5000× *g* for 10 min to obtain plasma. Plasma was stored at −20 °C for further characterisation. Hearts were then removed from rats for isolated Langendorff heart preparation [23].

Rats were orally gavaged with 3 mL of 0.05% phenol red solution 20 min before euthanasia. After euthanasia, the entire region from the stomach to small intestine was removed from its mesenteric attachment immediately. The length of small intestine was measured from the pyloric sphincter to the ileocecal junction. The endpoint of phenol red transit in the small intestine was visualised using a few drops of 0.1 M sodium hydroxide [77]. The % intestinal transit for each rat was determined by the following formula:

Intestinal transit (%)=(the total distance travelled by phenol red solution/total length of small intestine)×100

After isolated heart perfusion studies, the heart was separated into left ventricle (with septum) and right ventricle and weighed. The liver was isolated and weighed. Retroperitoneal, epididymal and omental fat pads were removed separately and weighed. These organ weights were normalised against tibial length at the time of organ removal and expressed as mg/mm of tibial length [23].

Hearts and livers (*n* = 4 from each group) were removed from the rats soon after euthanasia and fixed in 10% neutral buffered formalin. These samples were then dehydrated and embedded in paraffin wax. Thin sections (5 µm) were cut and stained with haematoxylin and eosin to study infiltration of inflammatory cells (heart and liver) and fat deposition (liver) and with picrosirius red (heart) to study collagen deposition [23].

Plasma activities of aspartate transaminase, alanine transaminase and alkaline phosphatase, and plasma concentrations of total cholesterol, triglycerides and non-esterified fatty acids were measured [23].

After euthanasia and organ removal, two or three faecal pellets were collected from the colon of rats and stored at −80 °C in nuclease-free tubes. DNA extraction and bacterial diversity profiling were performed by the Australian Genome Research Facility, Brisbane, QLD, Australia. The V_3_–V_4_ region of the 16S rRNA gene was selected for amplification. The detailed methods for this analysis were described in our previous study [13].

### 4.6. Stastical Analysis

All data are presented as mean ± SEM. Results were tested for variance using Bartlett’s test and variables that were not normally distributed were transformed (using log 10 functions) prior to statistical analyses. C, CCP, H and HCP groups were tested for effects of diet, treatment and their interactions by two-way analysis of variance. When the interaction and/or the main effects were significant, means were compared using Newman–Keuls multiple comparison post-hoc tests. A *p*-value of <0.05 was considered significant. All statistical analyses were performed using GraphPad Prism version 5.0 for Windows (San Diego, CA, USA). Microbiota data were analysed for statistical significance as detailed in a previous study [13]. Briefly, the V_3_–V_4_ region of the 16S rRNA gene were amplified and sequenced using the Illumina MiSeq platform. The resulting sequencing reads were quality filtered and zOTUs were generated using the UNOISE3 algorithm [78]. Chimeric sequences were removed and zOTU sequences were taxonomically assigned with the BLCA algorithm [79] against the Genome Taxonomy Database, which conservatively removes polyphyletic groups that are an issue in other taxonomic systems and normalises taxonomic ranks on the basis of relative evolutionary divergence [80]. A mapping of taxonomic names in the Genome Taxonomy Database within the National Centre of Biotechnology Information database can be found at: https://gtdb.ecogenomic.org/ (accessed on 15 July 2021).

To determine which zOTUs were affected by diet and supplementation, a two-factor design was used with the Multivariate Generalised Linear Models (MGLM) implemented in the package MVabund [81]. Each zOTU was treated as a variable fitted to a separate Generalised Linear Model (GLM) using a negative binomial distribution. An adjusted *p*-value of <0.05 was considered to be significant.

## 5. Conclusions

Coffee pulp is produced in large quantities around the world. This study provides initial evidence that coffee pulp can deliver similar results on both pathophysiology and metabolic variables as other products from coffee beans. Coffee pulp intervention in rats with diet-induced metabolic syndrome reduced plasma lipid concentrations, improved glucose tolerance and contributed to reduced obesity, dyslipidaemia and hyperglycaemia. With modulation of gut microbiota, coffee pulp has the potential to introduce similar changes in humans. Thus, developing products from coffee pulp could benefit people suffering from metabolic disorders. Human clinical trials are necessary to determine whether dietary coffee pulp supplementation can attenuate or reverse metabolic disorders associated with metabolic syndrome, particularly obesity, hypertension and fatty liver, with minimal adverse effects.

## Figures and Tables

**Table 1:** Intakes of components from coffee pulp.

Components	CCP	HCP
Caffeine, mg/kg/day	24.7 ± 0.5	17.1 ± 0.9
Chlorogenic acid, mg/kg/day	4.31 ± 0.08	2.98 ± 0.16
Phenolic acids, mg/kg/day	7.97 ± 0.15	5.51 ± 0.29
Trigonelline, mg/kg/day	14.6 ± 0.3	10.1 ± 0.5
Diterpenes, mg/kg/day	106.8 ± 2.0	73.9 ± 3.9

CCP, corn starch diet-fed rats supplemented with coffee pulp; HCP, high-carbohydrate, high-fat diet-fed rats supplemented with coffee pulp.

**Table 2:** Effects of coffee pulp on physiological parameters.

Variables	C	CCP	H	HCP	*p* Values		
					Diet	Treatment	Diet × Treatment
Initial body weight, g	339 ± 1	338 ± 1	340 ± 1	339 ± 1	1.00	0.32	0.32
Final body weight, g	380 ± 3 ^c^	386 ± 6 ^c^	551 ± 11 ^a^	494 ± 9 ^b^	<0.0001	0.0022	0.0002
Food intake, g/day	35.2 ± 1.3 ^a^	34.2 ± 0.6 ^a^	27.2 ± 0.7 ^b^	28.0 ± 1.5 ^b^	<0.0001	0.93	0.42
Water intake, g/day	31.0 ± 2.0 ^a,b^	36.8 ± 2.1 ^a^	26.6 ± 1.6 ^b^	32.2 ± 1.7 ^a,b^	0.02	0.004	0.96
Energy intake, kJ/d	405 ± 15 ^c^	384 ± 10 ^c^	556 ± 19 ^b^	616 ± 17 ^a^	<0.0001	0.22	0.013
Feed efficiency, g/kJ	0.10 ± 0.01 ^c^	0.12 ± 0.02 ^c^	0.36 ± 0.02 ^a^	0.25 ± 0.02 ^b^	<0.0001	0.016	0.0008
Heat, kcal/h	3.66 ± 0.25 ^b,c^	3.14 ± 0.31 ^c^	4.27 ± 0.16 ^a,b^	4.51 ± 0.16 ^a^	<0.0001	0.54	0.10
Respiratory exchange ratio	0.99 ± 0.02 ^a^	0.95 ± 0.04 ^a,b,c^	0.88 ± 0.02 ^c^	0.89 ± 0.01 ^b,c^	0.0014	0.55	0.32
Body mass index, g/cm^2^	0.61 ± 0.01 ^d^	0.66 ± 0.01 ^c^	0.83 ± 0.02 ^a^	0.75 ± 0.01 ^b^	<0.0001	0.26	<0.0001
Abdominal circumference, cm	18.8 ± 0.1 ^c^	17.8 ± 0.2 ^d^	23.7 ± 0.3 ^a^	20.2 ± 0.2 ^b^	<0.0001	<0.0001	<0.0001
Basal blood glucose, mmol/L	4.0 ± 0.2 ^b^	2.9 ± 0.1 ^c^	4.7 ± 0.3 ^a^	3.2 ± 0.1 ^c^	0.013	<0.0001	0.31
Area under the curve for glucose tolerance, mmol/L × min	695 ± 15 ^b^	421 ± 13 ^d^	797 ± 21 ^a^	571 ± 13 ^c^	<0.0001	<0.0001	0.14
Retroperitoneal fat, mg/mm	155 ± 14 ^b^	196 ± 14 ^b^	518 ± 42 ^a^	458 ± 22 ^a^	<0.0001	0.71	0.06
Epididymal fat, mg/mm	85 ± 5 ^c^	86 ± 10 ^c^	234 ± 17 ^a^	183 ± 10 ^b^	<0.0001	0.033	0.027
Omental fat, mg/mm	124 ± 7 ^c^	117 ± 11 ^c^	262 ± 12 ^a^	213 ± 12 ^b^	<0.0001	0.012	0.06
Total abdominal fat, mg/mm	364 ± 18 ^c^	399 ± 28 ^c^	1014 ± 65 ^a^	855 ± 33 ^b^	<0.0001	0.13	0.019
Whole-body lean mass, g	297 ± 4 ^a,b^	295 ± 4 ^a,b^	285 ± 8 ^b^	314 ± 7 ^a^	0.56	0.03	0.014
Whole-body fat mass, g	88 ± 6 ^c^	77 ± 7 ^c^	239 ± 12 ^a^	168 ± 11 ^b^	<0.0001	<0.0001	0.003
Bone mineral content, g	11.8 ± 0.4 ^b^	12.2 ± 0.5 ^b^	17.4 ± 0.6 ^a^	15.8 ± 0.5 ^a^	<0.0001	0.24	0.06
Bone mineral density, g/cm^2^	0.172 ± 0.002 ^c^	0.188 ± 0.002 ^b^	0.185 ± 0.002 ^b^	0.197 ± 0.005 ^a^	0.0002	<0.0001	0.47
Intestinal transit, %	74.1 ± 8.1 ^a,b^	52.2 ± 7.5 ^b^	90.2 ± 6.2 ^a^	58.2 ± 8.0 ^b^	0.15	0.0008	0.50
Plasma total cholesterol, mmol/L	1.79 ± 0.09 ^a^	0.56 ± 0.06 ^b^	1.56 ± 0.08 ^a^	1.84 ± 0.13 ^a^	<0.0001	<0.0001	<0.0001
Plasma triglycerides, mmol/L	0.57 ± 0.06 ^b^	0.33 ± 0.04 ^b^	1.28 ± 0.14 ^a^	0.61 ± 0.07 ^b^	<0.0001	<0.0001	0.016
Plasma non-esterified fatty acids, mmol/L	1.39 ± 0.18 ^b^	0.76 ± 0.14 ^c^	3.46 ± 0.22 ^a^	1.53 ± 0.20 ^b^	<0.0001	<0.0001	0.001
Systolic blood pressure, mmHg	127 ± 1 ^b^	125 ± 1 ^b^	143 ± 2 ^a^	129 ± 1 ^b^	<0.0001	<0.0001	<0.0001
Diastolic stiffness constant (κ)	21.9 ± 0.5 ^b^	20.5 ± 0.4 ^b^	29.3 ± 1.9 ^a^	21.0 ± 0.3 ^b^	<0.0001	0.0004	0.002
Left ventricle + septum wet weight, mg/mm	21.4 ± 0.8 ^b^	20.4 ± 0.7 ^b^	25.4 ± 1.5 ^a^	23.5 ± 0.8 ^a,b^	0.001	0.16	0.66
Right ventricle wet weight, mg/mm	4.82 ± 0.33	4.44 ± 0.20	5.42 ± 0.49	5.15 ± 0.20	0.051	0.33	0.87
Liver wet weight, mg/mm	227 ± 6 ^c^	245 ± 10 ^c^	349 ± 7 ^a^	293 ± 10 ^b^	<0.0001	0.029	<0.0001
Plasma aspartate transaminase activity, U/L	70.5 ± 3.3 ^c^	94.7 ± 3.9 ^a,b^	81.0 ± 5.4 ^b,c^	103.1 ± 6.7 ^a^	0.07	<0.0001	0.83
Plasma alanine transaminase activity, U/L	29.4 ± 4.2	37.4 ± 2.8	36.6 ± 3.6	42.1 ± 3.5	0.10	0.06	0.73

Values are mean ± SEM, *n* = 8–12. Means in a row with unlike superscripts (a, b, c or d) differ significantly, *p* < 0.05. C, corn starch diet-fed rats; CCP, corn starch diet-fed rats supplemented with coffee pulp; H, high-carbohydrate, high-fat diet-fed rats; HCP, high-carbohydrate, high-fat diet-fed rats supplemented with coffee pulp.

**Table 3:** Correlation between bacterial community structure and physiological parameters.

Physiological Variable	R^2^	*p* Value
Epididymal fat	0.74	0.001
Liver wet weight	0.71	0.001
Omental fat	0.64	0.001
Total abdominal fat	0.64	0.001
Retroperitoneal fat	0.57	0.001
Kidneys wet weight	0.49	0.001
Water intake	0.48	0.002
Alanine transaminase activity	0.47	0.002
Systolic blood pressure	0.40	0.005
Spleen wet weight	0.37	0.002
Feed efficiency	0.33	0.013
Non-esterified fatty acids	0.28	0.032
Body weight gain	0.28	0.030
Aspartate transaminase activity	0.26	0.042

## Data Availability

The data presented in this study are available on request from the corresponding author.

## Supplementary Materials

The following are available online at https://www.mdpi.com/article/10.3390/pathogens10111369/s1, Figure S1: HPLC-UV chromatogram top to bottom—254 nm for trigonelline, 280 nm for caffeine, 330 nm for chlorogenic acid and phenolic acids; Figure S2: (A) Trigonelline UV-Vis spectra λ max ~266 nm, (B) Caffeine UV-Vis spectra λ max ~274 nm, (C) Chlorogenic acid UV-Vis spectra λ max ~325 nm; Figure S3: HPLC-UV chromatogram 225 nm for diterpenes in coffee pulp; Figure S4: (A) Kahweol UV-Vis λ max ~290 nm, (B) Cafestol UV-Vis spectra λ max ~226 nm; Figure S5: Rarefaction curves; Figure S6: Shannon diversity (A) and richness (B) of faecal samples; Figure S7: Taxonomic profiles of bacterial communities shown at the class level of all faecal samples; Figure S8: Taxonomic profiles of bacterial communities shown at the family level of all faecal samples; Figure S9: nMDS plot of physiological data from 23 physiological parameters measured from different feeding regimes; Table S1: PERMANOVAs based on Bray–Curtis (BC) similarity measure for square-root transformed abundances of all rat faecal samples; Table S2: Differential zOTU abundance between C and H rats; Table S3: Differential zOTU abundance between C and CCP rats; Table S4: Differential zOTU abundance between H and HCP rats.
