# Supplementary material for: Coffee Pulp, a By-Product of Coffee Production, Modulates Gut Microbiota and Improves Metabolic Syndrome in High-Carbohydrate, High-Fat Diet-Fed Rats
Source: Pathogens. 2021 Oct 22;10(11):1369. doi: 10.3390/pathogens10111369 (PMC8624503; doi:10.3390/pathogens10111369)
Supplement: Supplementary file 1 [file pathogens-10-01369-s001.zip › pathogens-1386393-supplementary.pdf]

pathogens

MDPI

Article

Coffee Pulp, a By-Product of Coffee Production, Modulates Gut Microbiota and Improves Metabolic Syndrome in High-Carbohydrate, High-Fat Diet-Fed Rats

Nikhil S. Bhandarkar [1,†], Peter Mouatt [2], Marwan E. Majzoub [3], Torsten Thomas [3], Lindsay Brown [1,‡], and Sunil K. Panchal [1,*]

[1] Functional Foods Research Group, University of Southern Queensland, Toowoomba, QLD 4350, Australia; bhandark@post.bgu.ac.il (N.S.B); lindsay.brown@griffith.edu.au (L.B.)

[2] Southern Cross Plant Science, Southern Cross University, Lismore, NSW 2480, Australia; Peter.Mouatt@scu.edu.au

[3] Centre for Marine Science and Innovation & School of Biological, Earth and Environmental Sciences, University of New South Wales, Sydney, NSW 2052, Australia; m.majzoub@unsw.edu.au (M.E.M.); t.thomas@unsw.edu.au (T.T.)

* Correspondence: S.Panchal@westernsydney.edu.au; Tel.: +61-2-4570-1932; Present address: School of Science, Western Sydney University, Richmond, NSW 2753, Australia

† Present address: Department of Clinical Biochemistry and Pharmacology, Ben-Gurion University of the Negev, Beer-Sheva, Israel

‡ Present address: School of Pharmacy and Medical Science, Griffith University, Southport, QLD 4222, Australia

Citation: Bhandarkar, N.S.; Mouatt, P.; Majzoub, M.E.; Thomas, T.; Brown, L.; Panchal, S.K. Coffee Pulp, a By-Product of Coffee Production, Modulates Gut Microbiota and Improves Metabolic Syndrome in High-Carbohydrate, High-Fat Diet-Fed Rats. *Pathogens* **2021**, *10*, x. https://doi.org/10.3390/xxxxx

Academic Editor: Ana Elena Pérez Cobas

Received: 2 September 2021

Accepted: 18 October 2021

Published: date

Publisher's Note: MDPI stays neutral with regard to jurisdictional claims in published maps and institutional affiliations.

Copyright: © 2021 by the authors. Submitted for possible open access publication under the terms and conditions of the Creative Commons Attribution (CC BY) license (https://creativecommons.org/licenses/by/4.0/).

Supplementary Information

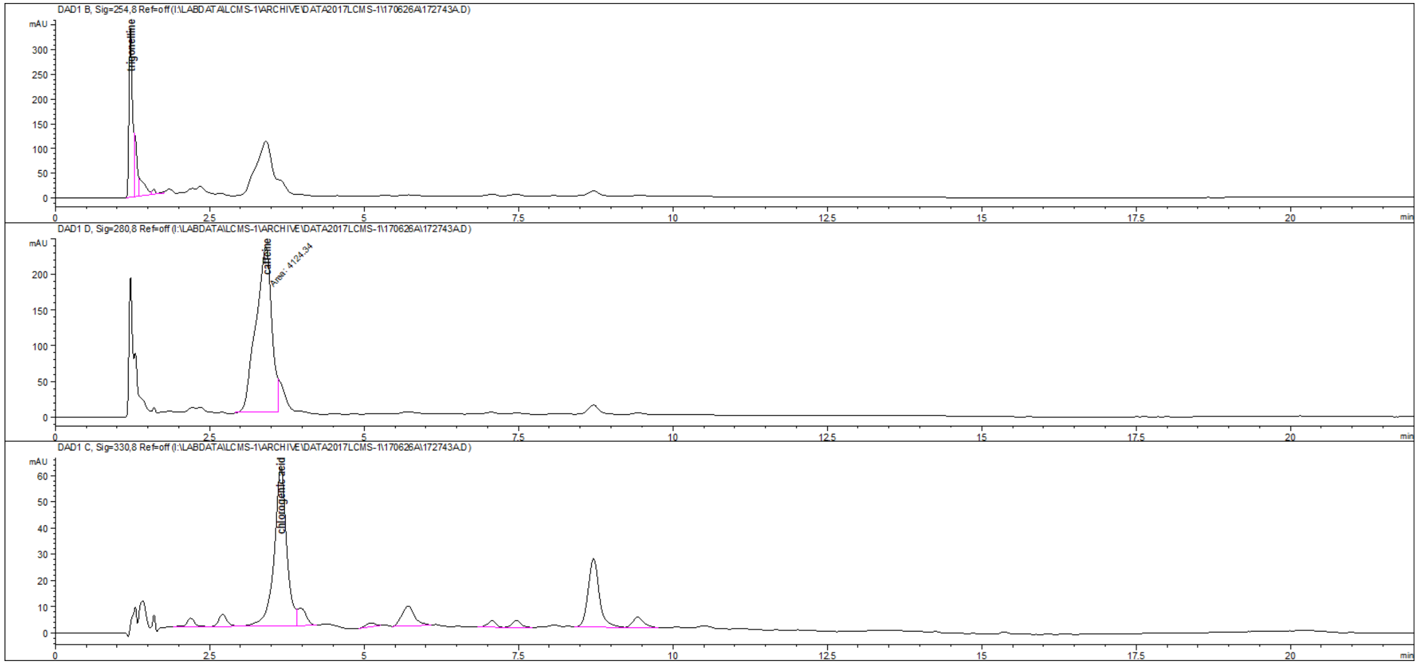

Figure S1. HPLC-UV chromatogram top to bottom – 254nm for trigonelline, 280nm for caffeine, 330nm for chlorogenic acid and phenolic acids.

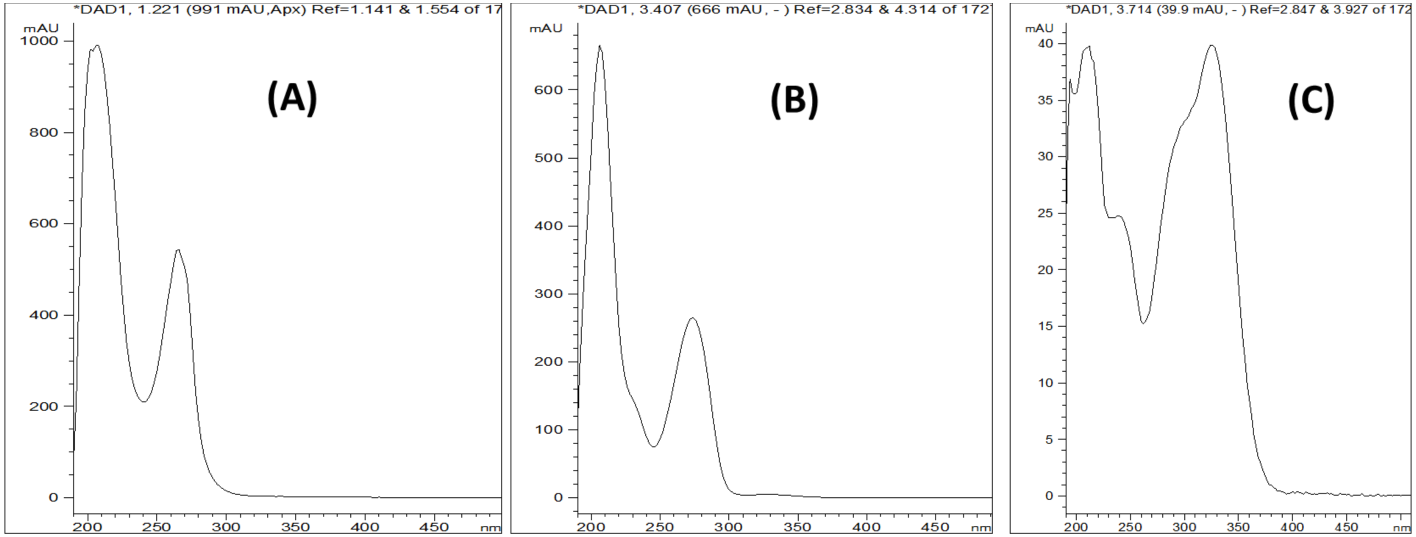

Figure S2. (A) Trigonelline UV-Vis spectra λ max ~266nm, **(B)** Caffeine UV-Vis spectra λ max ~274nm, **(C)** Chlorogenic acid UV-Vis spectra λ max ~325nm.

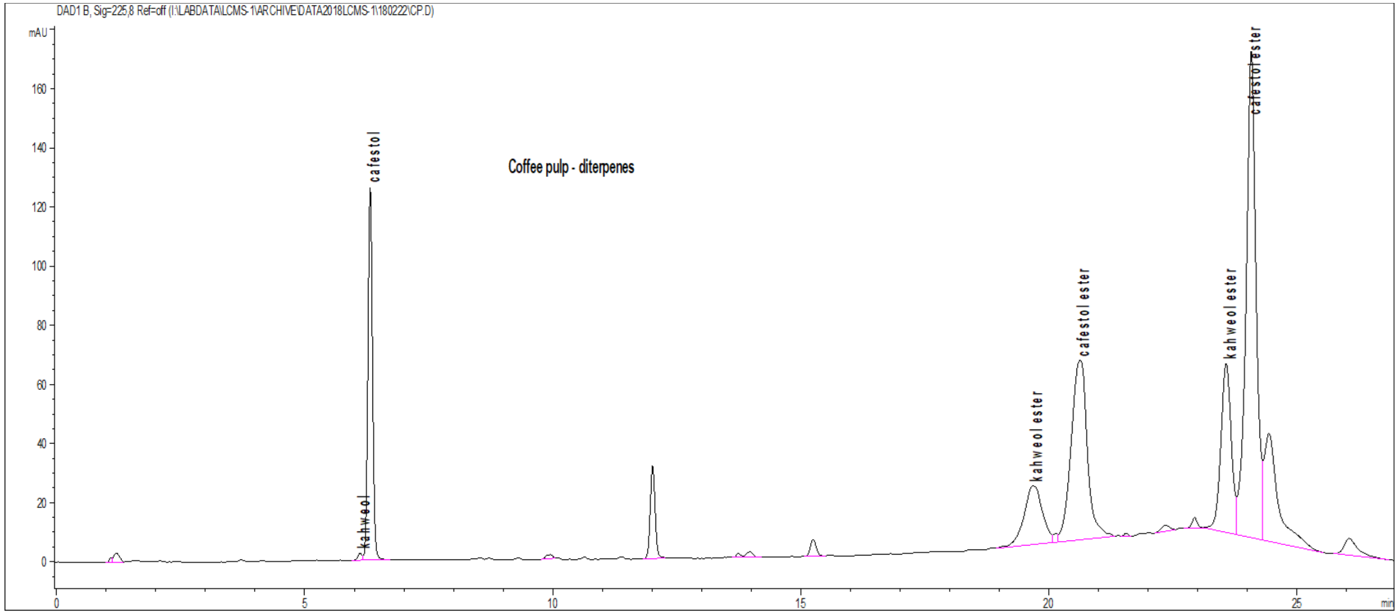

Figure S3. HPLC-UV chromatogram 225nm for diterpenes in coffee pulp [74,75].

•

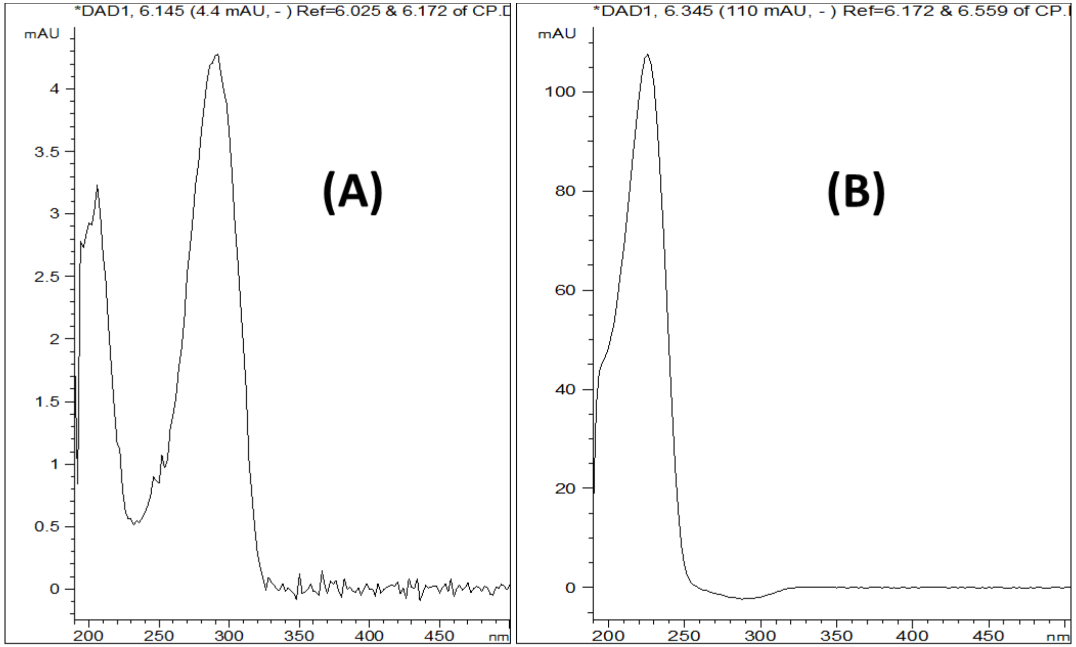

Figure S4. (A) Kahweol UV-Vis λ max ~290 nm, **(B)** Cafestol UV-Vis spectra λ max ~226 nm.

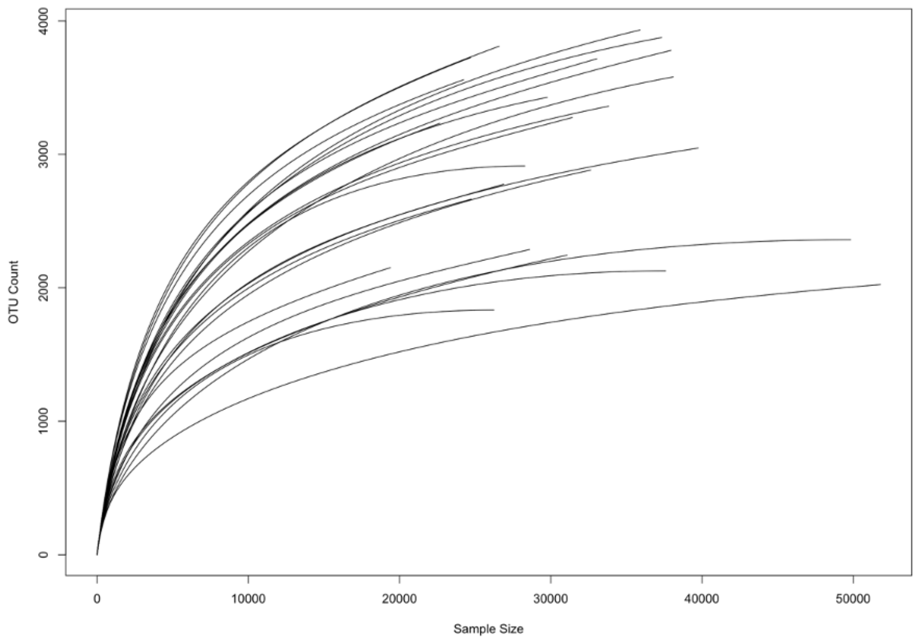

Figure S5. Rarefaction curves. 16S rRNA gene-based analysis was used to assess the bacterial communities from a total of 24 rat faecal pellets (6 rats per group). After quality filtering, there were a total of 772,429 sequences and these were clustered into 6,427 zOTUs. The calculated rarefaction curves based on rarefied and unrarefied data as well as Good's coverage of 85.8 ± 25.5% indicating that the majority of the bacterial community was recovered by the surveying effort.

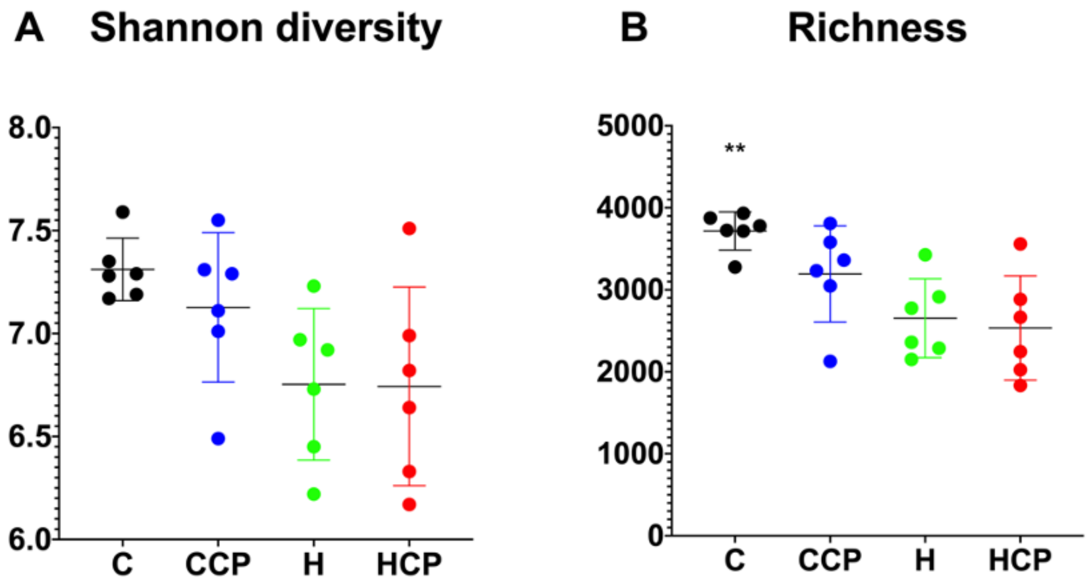

Figure S6. Shannon diversity (**A**) and richness (**B**) of faecal samples. ** vs CCP, H and HCP, p <0.05.

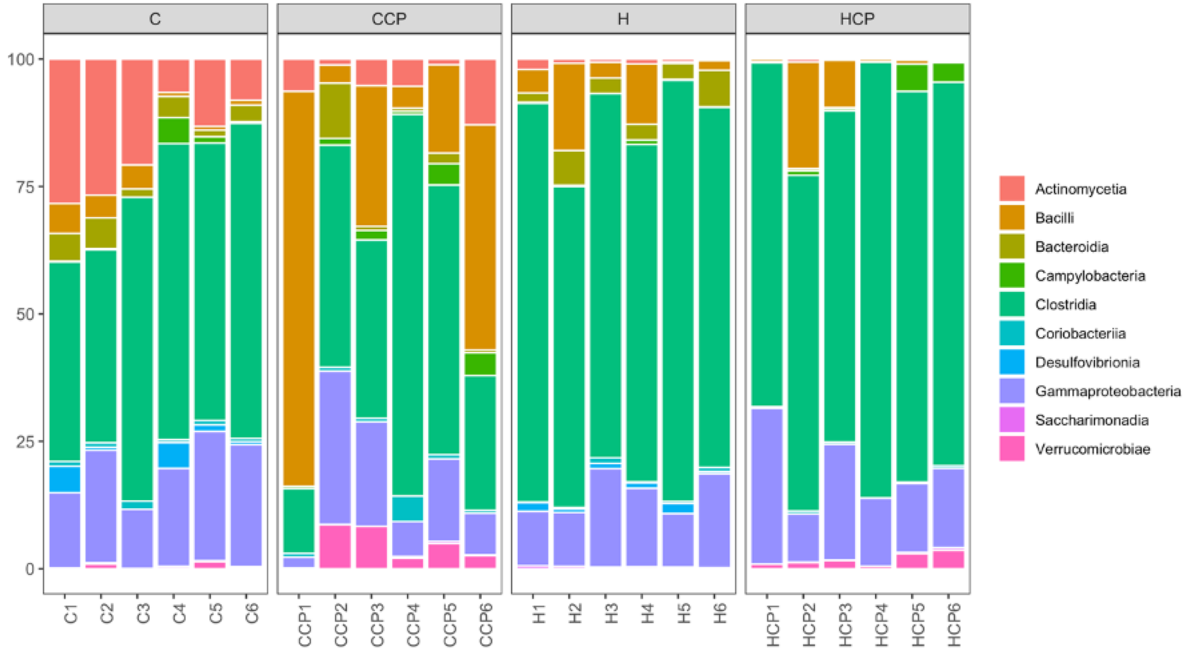

Figure S7. Taxonomic profiles of bacterial communities shown at the class level of all faecal samples.

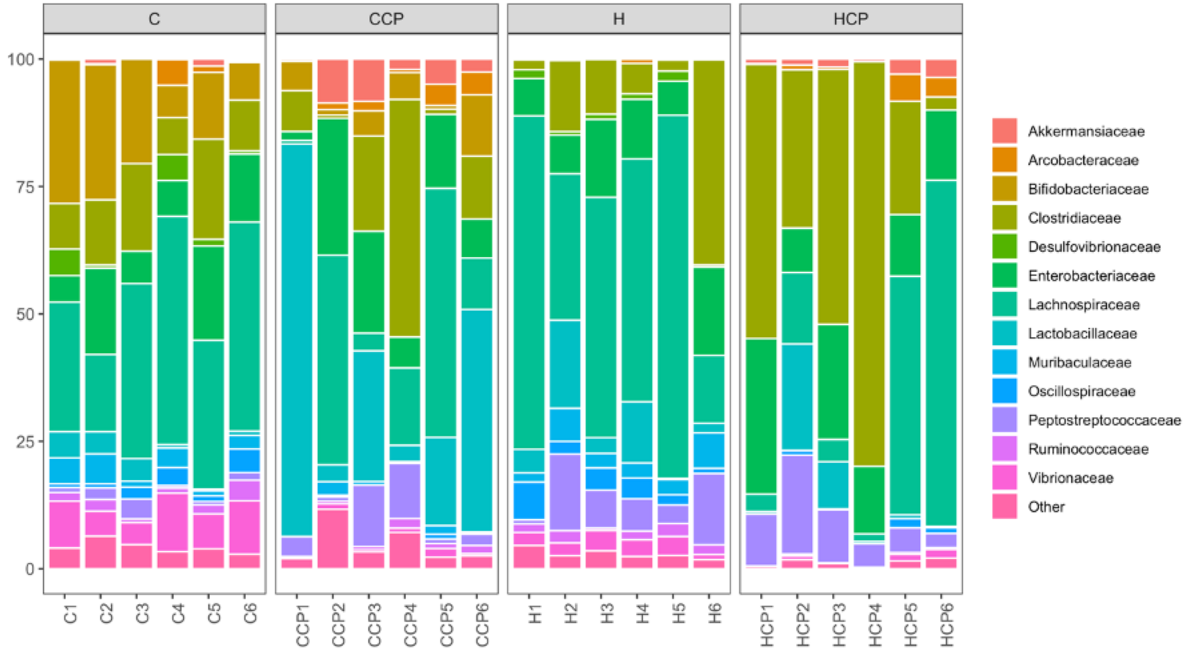

Figure S8. Taxonomic profiles of bacterial communities shown at the family level of all faecal samples.

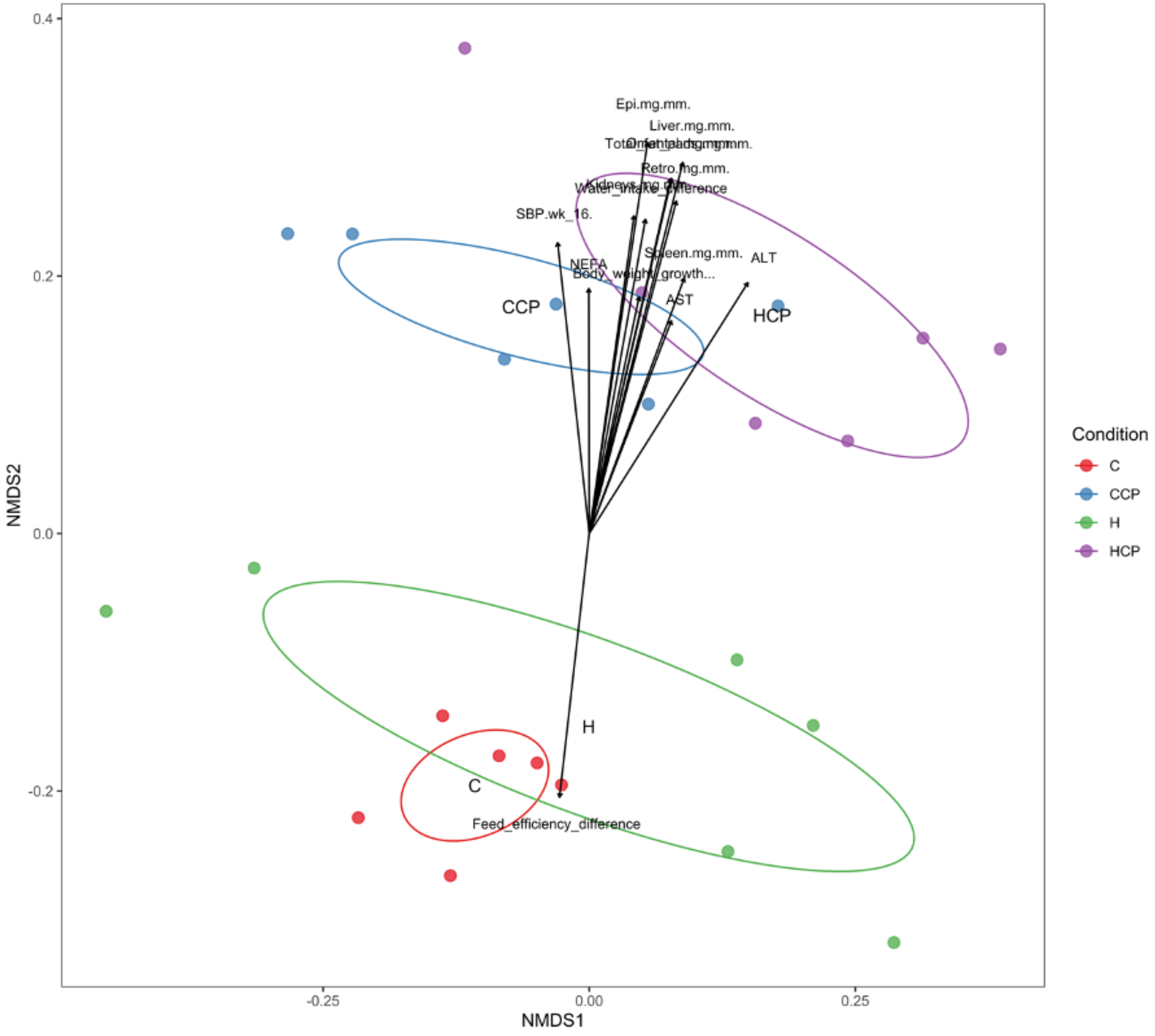

Figure S9. nMDS plot of physiological data from 23 physiological parameters measured from different feeding regimes.

Table S1. PERMANOVAs based on Bray-Curtis (BC) similarity measure for square-root transformed abundances of all rat faecal samples.

Source	df	Sums of Sqs	MeanSqs	Pseudo-F	*p*
Diet	1	1.002	1.00197	5.54027	0.0001
Treatment	2	0.9923	0.9923	5.3506	0.0001
Diet × treatment	2	0.4138	0.41377	2.2311	0.017
Res	20	3.7091	0.18546		
Total	23	6.1172			
**PAIR-WISE TESTS**					
**Groups**				***F.model***	***p***
C, CCP				3.999212	0.024
C, H				4.762622	0.012
C, HCP				6.094594	0.018
CCP, H				4.777236	0.03
CCP, HCP				3.147970	0.066
H, HCP				3.577782	0.108

P-values were calculated using 9,999 permutations under a residual model ($p < 0.05$). C, corn starch diet-fed rats; CCP, corn starch diet-fed rats supplemented with coffee pulp; H, high-carbohydrate, high-fat diet-fed rats; HCP, high-carbohydrate, high-fat diet-fed rats supplemented with coffee pulp.

Table S2. Differential zOTU abundance between C and H rats.

zOTU_ID	H%	HCP%	Phylum	Class	Family	Genus	Species
Zotu5206	0.00	0.08	Actinobacteriota	Coriobacteriia	*Eggerthellaceae*	*CAG-1427*	*CAG-1427 sp000435675*
Zotu5890	0.03	0.00	Bacteroidota	Bacteroidia	*Muribaculaceae*	*Muribaculum*	*Muribaculum sp001701195*
Zotu5281	0.08	0.00	Bacteroidota	Bacteroidia	*Muribaculaceae*	*Muribaculum*	*Muribaculum sp001701195*
Zotu4405	0.05	0.00	Bacteroidota	Bacteroidia	*Muribaculaceae*	*Muribaculum*	*Muribaculum sp001701195*
Zotu5561	0.11	0.00	Bacteroidota	Bacteroidia	*Muribaculaceae*	*Muribaculum*	*Muribaculum sp002492595*
Zotu4473	0.14	0.00	Bacteroidota	Bacteroidia	*Muribaculaceae*	*Muribaculum*	*Muribaculum sp002492595*
Zotu5048	0.06	0.00	Bacteroidota	Bacteroidia	*Muribaculaceae*	*Muribaculum*	*Muribaculum sp002492595*
Zotu4025	0.04	0.00	Bacteroidota	Bacteroidia	*Muribaculaceae*	*Muribaculum*	*Muribaculum sp002492595*
Zotu5809	0.12	0.00	Bacteroidota	Bacteroidia	*Muribaculaceae*	*Muribaculum*	*Muribaculum sp002492595*
Zotu4089	0.17	0.00	Bacteroidota	Bacteroidia	*Muribaculaceae*	*Muribaculum*	*Muribaculum sp002492595*
Zotu4227	0.09	0.00	Bacteroidota	Bacteroidia	*Muribaculaceae*	*Muribaculum*	*Muribaculum sp002492595*
Zotu5217	0.06	0.00	Bacteroidota	Bacteroidia	*Muribaculaceae*	*Muribaculum*	*Muribaculum sp002492595*
Zotu5577	0.05	0.00	Bacteroidota	Bacteroidia	*Muribaculaceae*	*Muribaculum*	*Muribaculum sp002492595*
Zotu4197	0.18	0.00	Bacteroidota	Bacteroidia	*Muribaculaceae*	*Muribaculum*	*Muribaculum sp002492595*
Zotu2681	0.11	0.00	Bacteroidota	Bacteroidia	*Muribaculaceae*	*Muribaculum*	*Muribaculum sp002492595*
Zotu3028	0.06	0.00	Bacteroidota	Bacteroidia	*Muribaculaceae*	*Muribaculum*	*Muribaculum sp002492595*
Zotu4991	0.04	0.00	Bacteroidota	Bacteroidia	*Muribaculaceae*	*Muribaculum*	*Muribaculum sp002492595*
Zotu6229	0.02	0.00	Bacteroidota	Bacteroidia	*Muribaculaceae*	*Muribaculum*	*Muribaculum sp002492595*
Zotu6469	0.03	0.00	Bacteroidota	Bacteroidia	*Muribaculaceae*	*Muribaculum*	*Muribaculum sp002492595*
Zotu6156	0.10	0.00	Bacteroidota	Bacteroidia	*Muribaculaceae*	*Muribaculum*	*Muribaculum sp002492595*
Zotu2423	0.04	0.00	Bacteroidota	Bacteroidia	*Muribaculaceae*	*Muribaculum*	*Muribaculum sp002492595*
Zotu4819	0.07	0.00	Bacteroidota	Bacteroidia	*Muribaculaceae*	*Muribaculum*	*Muribaculum sp002492595*
Zotu5215	0.04	0.00	Bacteroidota	Bacteroidia	*Muribaculaceae*	*Muribaculum*	*Muribaculum sp002492595*
Zotu2528	0.04	0.00	Bacteroidota	Bacteroidia	*Muribaculaceae*	*Muribaculum*	*Muribaculum sp002492595*
Zotu5445	0.02	0.00	Bacteroidota	Bacteroidia	*Muribaculaceae*	*Muribaculum*	*Muribaculum sp002492595*
Zotu5255	0.08	0.00	Bacteroidota	Bacteroidia	*Muribaculaceae*	*Muribaculum*	*Muribaculum sp002492595*
Zotu5862	0.03	0.00	Bacteroidota	Bacteroidia	*Muribaculaceae*	*Muribaculum*	*Muribaculum sp002492595*
Zotu4527	0.06	0.00	Bacteroidota	Bacteroidia	*Muribaculaceae*	*Muribaculum*	*Muribaculum sp002492595*
Zotu6607	0.07	0.00	Bacteroidota	Bacteroidia	*Muribaculaceae*	*Muribaculum*	*Muribaculum sp002492595*
Zotu3584	0.03	0.00	Bacteroidota	Bacteroidia	*Muribaculaceae*	*Muribaculum*	*Muribaculum sp002492595*
Zotu6093	0.03	0.00	Bacteroidota	Bacteroidia	*Muribaculaceae*	*Muribaculum*	*Muribaculum sp002492595*
Zotu6638	0.07	0.00	Bacteroidota	Bacteroidia	*Muribaculaceae*	*Muribaculum*	*Muribaculum sp002492595*
Zotu4859	0.04	0.00	Bacteroidota	Bacteroidia	*Muribaculaceae*	*Muribaculum*	*Muribaculum sp002492595*
Zotu5152	0.03	0.00	Bacteroidota	Bacteroidia	*Muribaculaceae*	*Muribaculum*	*Muribaculum sp002492595*

Zotu6493	0.02	0.00	Bacteroidota	Bacteroidia	*Muribaculaceae*	*Muribaculum*	*Muribaculum sp002492595*
Zotu6097	0.03	0.00	Bacteroidota	Bacteroidia	*Muribaculaceae*	*Muribaculum*	*Muribaculum sp002492595*
Zotu5484	0.04	0.00	Bacteroidota	Bacteroidia	*Muribaculaceae*	*Muribaculum*	*Muribaculum sp002492595*
Zotu5534	0.02	0.00	Bacteroidota	Bacteroidia	*Muribaculaceae*	*Muribaculum*	*Muribaculum sp002492595*
Zotu6489	0.03	0.00	Bacteroidota	Bacteroidia	*Muribaculaceae*	*Muribaculum*	*Muribaculum sp002492595*
Zotu6460	0.02	0.00	Bacteroidota	Bacteroidia	*Muribaculaceae*	*Muribaculum*	*Muribaculum sp002492595*
Zotu5572	0.02	0.00	Bacteroidota	Bacteroidia	*Muribaculaceae*	*Muribaculum*	*Muribaculum sp002492595*
Zotu6595	0.04	0.00	Bacteroidota	Bacteroidia	*Muribaculaceae*	*Muribaculum*	*Muribaculum sp002492595*
Zotu5658	0.02	0.00	Bacteroidota	Bacteroidia	*Muribaculaceae*	*Muribaculum*	*Muribaculum sp002492595*
Zotu5634	0.02	0.00	Bacteroidota	Bacteroidia	*Muribaculaceae*	*Muribaculum*	*Muribaculum sp002492595*
Zotu5807	0.03	0.00	Bacteroidota	Bacteroidia	*Muribaculaceae*	*Muribaculum*	*Muribaculum sp002492595*
Zotu5716	0.02	0.00	Bacteroidota	Bacteroidia	*Muribaculaceae*	*Muribaculum*	*Muribaculum sp002492595*
Zotu5951	0.02	0.00	Bacteroidota	Bacteroidia	*Muribaculaceae*	*Muribaculum*	*Muribaculum sp002492595*
Zotu3894	0.04	0.00	Bacteroidota	Bacteroidia	*Muribaculaceae*	*Muribaculum*	*Muribaculum sp002492595*
Zotu5743	0.02	0.00	Bacteroidota	Bacteroidia	*Muribaculaceae*	*Muribaculum*	*Muribaculum sp002492595*
Zotu4941	0.07	0.00	Bacteroidota	Bacteroidia	*Muribaculaceae*	*Muribaculum*	*Vibrio parahaemolyticus*
Zotu5804	0.04	0.00	Bacteroidota	Bacteroidia	*Muribaculaceae*	*UBA7173*	*UBA7173 sp004102805*
Zotu4753	0.05	0.00	Bacteroidota	Bacteroidia	*Muribaculaceae*	*UBA7173*	*UBA7173 sp004102805*
Zotu6105	0.05	0.00	Bacteroidota	Bacteroidia	*Muribaculaceae*	*Vibrio*	*Vibrio parahaemolyticus*
Zotu6700	0.03	0.00	Bacteroidota	Bacteroidia	*Muribaculaceae*	*Vibrio*	*Vibrio parahaemolyticus*
Zotu4475	0.05	0.00	Bacteroidota	Bacteroidia	*Muribaculaceae*	*Vibrio*	*Vibrio parahaemolyticus*
Zotu5865	0.03	0.00	Desulfobacterota	Desulfovibrionia	*Desulfovibrionaceae*	*Desulfovibrio*	*Desulfovibrio sp003860215*
Zotu3742	0.06	0.00	Desulfobacterota	Desulfovibrionia	*Desulfovibrionaceae*	*Desulfovibrio*	*Desulfovibrio sp003860215*
Zotu5370	0.02	0.00	Desulfobacterota	Desulfovibrionia	*Desulfovibrionaceae*	*Desulfovibrio*	*Desulfovibrio sp003860215*
Zotu3774	0.02	0.00	Desulfobacterota	Desulfovibrionia	*Desulfovibrionaceae*	*Desulfovibrio*	*Desulfovibrio sp003860215*
Zotu3238	0.02	0.00	Desulfobacterota	Desulfovibrionia	*Desulfovibrionaceae*	*Desulfovibrio*	*Desulfovibrio sp900547595*
Zotu4155	0.05	0.00	Desulfobacterota	Desulfovibrionia	*Desulfovibrionaceae*	*Desulfovibrio*	*Desulfovibrio sp900547595*
Zotu5342	0.01	0.00	Desulfobacterota	Desulfovibrionia	*Desulfovibrionaceae*	*Desulfovibrio*	*Desulfovibrio sp900547595*
Zotu3812	0.04	0.00	Desulfobacterota	Desulfovibrionia	*Desulfovibrionaceae*	*Desulfovibrio*	*Desulfovibrio sp900547595*
Zotu4998	0.02	0.00	Desulfobacterota	Desulfovibrionia	*Desulfovibrionaceae*	*Desulfovibrio*	*Desulfovibrio sp900547595*
Zotu4009	0.03	0.00	Desulfobacterota	Desulfovibrionia	*Desulfovibrionaceae*	*Desulfovibrio*	*Desulfovibrio sp900547595*
Zotu6629	0.02	0.00	Desulfobacterota	Desulfovibrionia	*Desulfovibrionaceae*	*Desulfovibrio*	*Desulfovibrio sp900547595*
Zotu4327	0.02	0.00	Desulfobacterota	Desulfovibrionia	*Desulfovibrionaceae*	*Desulfovibrio*	*Desulfovibrio sp900547595*
Zotu4782	0.03	0.00	Desulfobacterota	Desulfovibrionia	*Desulfovibrionaceae*	*Desulfovibrio*	*Desulfovibrio sp900547595*
Zotu4439	0.05	0.00	Desulfobacterota	Desulfovibrionia	*Desulfovibrionaceae*	*Desulfovibrio*	*Desulfovibrio sp900547595*
Zotu6265	0.02	0.00	Desulfobacterota	Desulfovibrionia	*Desulfovibrionaceae*	*Desulfovibrio*	*Desulfovibrio sp900547595*
Zotu4964	0.01	0.00	Desulfobacterota	Desulfovibrionia	*Desulfovibrionaceae*	*Desulfovibrio*	*Desulfovibrio sp900547595*

Zotu5179	0.00	0.03	Firmicutes A	Clostridia	*Clostridiaceae*	*Clostridium*	*Clostridium saudiense*
Zotu6695	0.00	0.21	Firmicutes A	Clostridia	*Lachnospiraceae*	*Bacteroides F*	*Bacteroides_F pectinophilus*
Zotu5489	0.00	0.46	Firmicutes A	Clostridia	*Lachnospiraceae*	*Bacteroides F*	*Bacteroides_F pectinophilus*
Zotu5932	0.00	0.14	Firmicutes A	Clostridia	*Lachnospiraceae*	*Bacteroides F*	*Bacteroides_F pectinophilus*
Zotu6424	0.00	0.23	Firmicutes A	Clostridia	*Lachnospiraceae*	*Bacteroides F*	*Bacteroides_F pectinophilus*
Zotu4341	0.00	0.29	Firmicutes A	Clostridia	*Lachnospiraceae*	*Bacteroides F*	*Bacteroides_F pectinophilus*
Zotu6321	0.80	0.00	Firmicutes A	Clostridia	*Lachnospiraceae*	*Enterocloster*	*Acetatifactor sp900066565*
Zotu6659	0.01	0.00	Firmicutes A	Clostridia	*Lachnospiraceae*	*Kineothrix*	*Kineothrix alysoides*
Zotu1092	0.00	0.04	Proteobacteria	Gammaproteobacteria	*Enterobacteriaceae*	*Cronobacter*	*Akkermansia muciniphila*
Zotu1361	0.00	0.02	Proteobacteria	Gammaproteobacteria	*Enterobacteriaceae*	*Cronobacter*	*Akkermansia muciniphila*
Zotu682	0.00	0.03	Proteobacteria	Gammaproteobacteria	*Enterobacteriaceae*	*Cronobacter*	*Akkermansia muciniphila*
Zotu627	0.00	0.03	Proteobacteria	Gammaproteobacteria	*Enterobacteriaceae*	*Cronobacter*	*Cronobacter malonaticus*
Zotu832	0.00	0.02	Proteobacteria	Gammaproteobacteria	*Enterobacteriaceae*	*Cronobacter*	*Cronobacter malonaticus*
Zotu861	0.00	0.01	Proteobacteria	Gammaproteobacteria	*Enterobacteriaceae*	*Cronobacter*	*Cronobacter malonaticus*
Zotu773	0.00	0.01	Proteobacteria	Gammaproteobacteria	*Enterobacteriaceae*	*Cronobacter*	*Cronobacter malonaticus*
Zotu1665	0.00	0.02	Proteobacteria	Gammaproteobacteria	*Enterobacteriaceae*	*Cronobacter*	*Cronobacter malonaticus*
Zotu595	0.00	0.08	Proteobacteria	Gammaproteobacteria	*Enterobacteriaceae*	*Cronobacter*	*Cronobacter malonaticus*
Zotu1458	0.00	0.01	Proteobacteria	Gammaproteobacteria	*Enterobacteriaceae*	*Cronobacter*	*Cronobacter malonaticus*
Zotu445	0.00	0.09	Proteobacteria	Gammaproteobacteria	*Enterobacteriaceae*	*Cronobacter*	*Cronobacter malonaticus*
Zotu352	0.00	0.06	Proteobacteria	Gammaproteobacteria	*Enterobacteriaceae*	*Cronobacter*	*Cronobacter malonaticus*
Zotu1176	0.00	0.01	Proteobacteria	Gammaproteobacteria	*Enterobacteriaceae*	*Cronobacter*	*Cronobacter malonaticus*
Zotu1279	0.00	0.04	Proteobacteria	Gammaproteobacteria	*Enterobacteriaceae*	*Cronobacter*	*Cronobacter malonaticus*

Zotu827	0.00	0.02	Proteobacteria	Gammaproteobacteria	*Enterobacteriaceae*	*Cronobacter*	*Cronobacter malonaticus*
Zotu610	0.00	0.03	Proteobacteria	Gammaproteobacteria	*Enterobacteriaceae*	*Cronobacter*	*Cronobacter malonaticus*
Zotu818	0.00	0.02	Proteobacteria	Gammaproteobacteria	*Enterobacteriaceae*	*Cronobacter*	*Cronobacter malonaticus*
Zotu931	0.00	0.02	Proteobacteria	Gammaproteobacteria	*Enterobacteriaceae*	*Cronobacter*	*Cronobacter malonaticus*
Zotu1470	0.00	0.01	Proteobacteria	Gammaproteobacteria	*Enterobacteriaceae*	*Cronobacter*	*Cronobacter malonaticus*
Zotu1322	0.00	0.01	Proteobacteria	Gammaproteobacteria	*Enterobacteriaceae*	*Cronobacter*	*Cronobacter malonaticus*
Zotu1822	0.00	0.01	Proteobacteria	Gammaproteobacteria	*Enterobacteriaceae*	*Cronobacter*	*Cronobacter malonaticus*
Zotu406	0.00	0.03	Proteobacteria	Gammaproteobacteria	*Enterobacteriaceae*	*Cronobacter*	*Cronobacter malonaticus*
Zotu855	0.00	0.03	Proteobacteria	Gammaproteobacteria	*Enterobacteriaceae*	*Cronobacter*	*Cronobacter sakazakii*
Zotu1009	0.00	0.02	Proteobacteria	Gammaproteobacteria	*Enterobacteriaceae*	*Cronobacter*	*Cronobacter sakazakii*
Zotu918	0.00	0.03	Proteobacteria	Gammaproteobacteria	*Enterobacteriaceae*	*Cronobacter*	*Cronobacter sakazakii*
Zotu428	0.00	0.02	Proteobacteria	Gammaproteobacteria	*Enterobacteriaceae*	*Cronobacter*	*Cronobacter sakazakii*
Zotu1658	0.00	0.03	Proteobacteria	Gammaproteobacteria	*Enterobacteriaceae*	*Cronobacter*	*Cronobacter sakazakii*
Zotu765	0.00	0.02	Proteobacteria	Gammaproteobacteria	*Enterobacteriaceae*	*Cronobacter*	*Cronobacter sakazakii*
Zotu888	0.00	0.01	Proteobacteria	Gammaproteobacteria	*Enterobacteriaceae*	*Cronobacter*	*Cronobacter sakazakii*
Zotu1081	0.00	0.01	Proteobacteria	Gammaproteobacteria	*Enterobacteriaceae*	*Cronobacter*	*Cronobacter sakazakii*
Zotu781	0.00	0.01	Proteobacteria	Gammaproteobacteria	*Enterobacteriaceae*	*Cronobacter*	*Cronobacter sakazakii*
Zotu720	0.00	0.07	Proteobacteria	Gammaproteobacteria	*Enterobacteriaceae*	*Cronobacter*	*Cronobacter sakazakii*

Zotu878	0.00	0.06	Proteobacteria	Gammaproteobacteria	*Enterobacteriaceae*	*Cronobacter*	*Cronobacter sakazakii*
Zotu1000	0.00	0.01	Proteobacteria	Gammaproteobacteria	*Enterobacteriaceae*	*Cronobacter*	*Cronobacter sakazakii*
Zotu1316	0.00	0.01	Proteobacteria	Gammaproteobacteria	*Enterobacteriaceae*	*Cronobacter*	*Cronobacter sakazakii*
Zotu1523	0.00	0.01	Proteobacteria	Gammaproteobacteria	*Enterobacteriaceae*	*Cronobacter*	*Cronobacter sakazakii*
Zotu706	0.00	0.03	Proteobacteria	Gammaproteobacteria	*Enterobacteriaceae*	*Cronobacter*	*Cronobacter sakazakii*
Zotu1909	0.00	0.01	Proteobacteria	Gammaproteobacteria	*Enterobacteriaceae*	*Cronobacter*	*Cronobacter sakazakii*
Zotu1152	0.00	0.02	Proteobacteria	Gammaproteobacteria	*Enterobacteriaceae*	*Cronobacter*	*Cronobacter sakazakii*
Zotu1367	0.00	0.02	Proteobacteria	Gammaproteobacteria	*Enterobacteriaceae*	*Cronobacter*	*Cronobacter sakazakii*
Zotu1720	0.00	0.01	Proteobacteria	Gammaproteobacteria	*Enterobacteriaceae*	*Cronobacter*	*Cronobacter sakazakii*
Zotu381	0.00	0.05	Proteobacteria	Gammaproteobacteria	*Enterobacteriaceae*	*Cronobacter*	*Cronobacter sakazakii*
Zotu736	0.00	0.04	Proteobacteria	Gammaproteobacteria	*Enterobacteriaceae*	*Cronobacter*	*Cronobacter sakazakii*
Zotu1077	0.00	0.04	Proteobacteria	Gammaproteobacteria	*Enterobacteriaceae*	*Cronobacter*	*Cronobacter sakazakii*
Zotu997	0.00	0.01	Proteobacteria	Gammaproteobacteria	*Enterobacteriaceae*	*Cronobacter*	*Cronobacter sakazakii*
Zotu974	0.00	0.02	Proteobacteria	Gammaproteobacteria	*Enterobacteriaceae*	*Cronobacter*	*Cronobacter sakazakii*
Zotu1038	0.00	0.02	Proteobacteria	Gammaproteobacteria	*Enterobacteriaceae*	*Cronobacter*	*Cronobacter sakazakii*
Zotu1432	0.00	0.02	Proteobacteria	Gammaproteobacteria	*Enterobacteriaceae*	*Cronobacter*	*Cronobacter sakazakii*
Zotu726	0.00	0.02	Proteobacteria	Gammaproteobacteria	*Enterobacteriaceae*	*Cronobacter*	*Cronobacter sakazakii*
Zotu1293	0.00	0.01	Proteobacteria	Gammaproteobacteria	*Enterobacteriaceae*	*Cronobacter*	*Cronobacter sakazakii*

Zotu1773	0.00	0.01	Proteobacteria	Gammaproteobacteria	*Enterobacteriaceae*	*Cronobacter*	*Cronobacter sakazakii*
Zotu576	0.00	0.03	Proteobacteria	Gammaproteobacteria	*Enterobacteriaceae*	*Cronobacter*	*Cronobacter sakazakii*
Zotu821	0.00	0.01	Proteobacteria	Gammaproteobacteria	*Enterobacteriaceae*	*Cronobacter*	*Cronobacter sakazakii*
Zotu695	0.00	0.15	Verrucomicrobiota	Verrucomicrobiae	*Akkermansiaceae*	*Akkermansia*	*Akkermansia muciniphila*
Zotu1477	0.00	0.02	Verrucomicrobiota	Verrucomicrobiae	*Akkermansiaceae*	*Akkermansia*	*Akkermansia muciniphila*
Zotu920	0.00	0.04	Verrucomicrobiota	Verrucomicrobiae	*Akkermansiaceae*	*Akkermansia*	*Akkermansia muciniphila*
Zotu505	0.00	0.04	Verrucomicrobiota	Verrucomicrobiae	*Akkermansiaceae*	*Akkermansia*	*Akkermansia muciniphila*
Zotu1011	0.00	0.05	Verrucomicrobiota	Verrucomicrobiae	*Akkermansiaceae*	*Akkermansia*	*Akkermansia muciniphila*
Zotu721	0.00	0.05	Verrucomicrobiota	Verrucomicrobiae	*Akkermansiaceae*	*Akkermansia*	*Akkermansia muciniphila*
Zotu724	0.00	0.04	Verrucomicrobiota	Verrucomicrobiae	*Akkermansiaceae*	*Akkermansia*	*Akkermansia muciniphila*
Zotu801	0.00	0.02	Verrucomicrobiota	Verrucomicrobiae	*Akkermansiaceae*	*Akkermansia*	*Akkermansia muciniphila*
Zotu1102	0.00	0.04	Verrucomicrobiota	Verrucomicrobiae	*Akkermansiaceae*	*Akkermansia*	*Akkermansia muciniphila*
Zotu1115	0.00	0.02	Verrucomicrobiota	Verrucomicrobiae	*Akkermansiaceae*	*Akkermansia*	*Akkermansia muciniphila*
Zotu1330	0.00	0.07	Verrucomicrobiota	Verrucomicrobiae	*Akkermansiaceae*	*Akkermansia*	*Akkermansia muciniphila*
Zotu1416	0.00	0.01	Verrucomicrobiota	Verrucomicrobiae	*Akkermansiaceae*	*Akkermansia*	*Akkermansia muciniphila*
Zotu1395	0.00	0.01	Verrucomicrobiota	Verrucomicrobiae	*Akkermansiaceae*	*Akkermansia*	*Akkermansia muciniphila*
Zotu1133	0.00	0.02	Verrucomicrobiota	Verrucomicrobiae	*Akkermansiaceae*	*Akkermansia*	*Akkermansia muciniphila*
Zotu1400	0.00	0.03	Verrucomicrobiota	Verrucomicrobiae	*Akkermansiaceae*	*Akkermansia*	*Akkermansia muciniphila*
Zotu740	0.00	0.04	Verrucomicrobiota	Verrucomicrobiae	*Akkermansiaceae*	*Akkermansia*	*Akkermansia muciniphila*
Zotu758	0.00	0.10	Verrucomicrobiota	Verrucomicrobiae	*Akkermansiaceae*	*Akkermansia*	*Akkermansia muciniphila*
Zotu1389	0.00	0.02	Verrucomicrobiota	Verrucomicrobiae	*Akkermansiaceae*	*Akkermansia*	*Akkermansia muciniphila*
Zotu1231	0.00	0.04	Verrucomicrobiota	Verrucomicrobiae	*Akkermansiaceae*	*Akkermansia*	*Akkermansia muciniphila*
Zotu1346	0.00	0.01	Verrucomicrobiota	Verrucomicrobiae	*Akkermansiaceae*	*Akkermansia*	*Akkermansia muciniphila*
Zotu757	0.00	0.04	Verrucomicrobiota	Verrucomicrobiae	*Akkermansiaceae*	*Akkermansia*	*Akkermansia muciniphila*
Zotu2262	0.00	0.01	Verrucomicrobiota	Verrucomicrobiae	*Akkermansiaceae*	*Akkermansia*	*Akkermansia muciniphila*
Zotu1160	0.00	0.01	Verrucomicrobiota	Verrucomicrobiae	*Akkermansiaceae*	*Akkermansia*	*Akkermansia muciniphila*

Table S3. Differential zOTU abundance between C and CCP rats.

zOTU_ID	C %	CCP %	Phylum	Class	Family	Genus	Species
Zotu5347	0.01	0.00	Actinobacteriota	Actinomycetia	*Bifidobacteriaceae*	*Bifidobacterium*	*Bifidobacterium criceti*
Zotu764	0.06	0.01	Actinobacteriota	Actinomycetia	*Bifidobacteriaceae*	*Bifidobacterium*	*Bifidobacterium globosum*
Zotu2358	0.02	0.00	Actinobacteriota	Actinomycetia	*Bifidobacteriaceae*	*Bifidobacterium*	*Bifidobacterium globosum*
Zotu488	0.08	0.01	Actinobacteriota	Actinomycetia	*Bifidobacteriaceae*	*Bifidobacterium*	*Bifidobacterium globosum*
Zotu1253	0.04	0.00	Actinobacteriota	Actinomycetia	*Bifidobacteriaceae*	*Bifidobacterium*	*Bifidobacterium globosum*
Zotu5206	0.00	0.13	Actinobacteriota	Coriobacteriia	*Eggerthellaceae*	*CAG-1427*	*CAG-1427 sp000435675*
Zotu5037	0.26	0.00	Bacteroidota	Bacteroidia	*Muribaculaceae*	*Muribaculum*	*Muribaculum intestinale*
Zotu5281	0.11	0.00	Bacteroidota	Bacteroidia	*Muribaculaceae*	*Muribaculum*	*Muribaculum sp001701195*
Zotu4405	0.08	0.00	Bacteroidota	Bacteroidia	*Muribaculaceae*	*Muribaculum*	*Muribaculum sp001701195*
Zotu4819	0.04	0.00	Bacteroidota	Bacteroidia	*Muribaculaceae*	*Muribaculum*	*Muribaculum sp002492595*
Zotu2681	0.07	0.00	Bacteroidota	Bacteroidia	*Muribaculaceae*	*Muribaculum*	*Muribaculum sp002492595*
Zotu6607	0.04	0.00	Bacteroidota	Bacteroidia	*Muribaculaceae*	*Muribaculum*	*Muribaculum sp002492595*
Zotu4197	0.11	0.00	Bacteroidota	Bacteroidia	*Muribaculaceae*	*Muribaculum*	*Muribaculum sp002492595*
Zotu2676	0.03	0.00	Bacteroidota	Bacteroidia	*Muribaculaceae*	*Muribaculum*	*Muribaculum sp002492595*
Zotu4089	0.10	0.00	Bacteroidota	Bacteroidia	*Muribaculaceae*	*Muribaculum*	*Muribaculum sp002492595*
Zotu6095	0.02	0.00	Bacteroidota	Bacteroidia	*Muribaculaceae*	*Muribaculum*	*Muribaculum sp002492595*
Zotu5809	0.05	0.00	Bacteroidota	Bacteroidia	*Muribaculaceae*	*Muribaculum*	*Muribaculum sp002492595*
Zotu4060	0.01	0.00	Bacteroidota	Bacteroidia	*Muribaculaceae*	*Muribaculum*	*Muribaculum sp002492595*
Zotu5255	0.05	0.00	Bacteroidota	Bacteroidia	*Muribaculaceae*	*Muribaculum*	*Muribaculum sp002492595*
Zotu4473	0.09	0.00	Bacteroidota	Bacteroidia	*Muribaculaceae*	*Muribaculum*	*Muribaculum sp002492595*
Zotu5561	0.06	0.00	Bacteroidota	Bacteroidia	*Muribaculaceae*	*Muribaculum*	*Muribaculum sp002492595*
Zotu3028	0.04	0.00	Bacteroidota	Bacteroidia	*Muribaculaceae*	*Muribaculum*	*Muribaculum sp002492595*
Zotu5275	0.02	0.00	Bacteroidota	Bacteroidia	*Muribaculaceae*	*UBA7173*	*UBA7173 sp004102805*
Zotu4753	0.08	0.00	Bacteroidota	Bacteroidia	*Muribaculaceae*	*UBA7173*	*UBA7173 sp004102805*
Zotu5804	0.04	0.00	Bacteroidota	Bacteroidia	*Muribaculaceae*	*UBA7173*	*UBA7173 sp004102805*
Zotu4475	0.08	0.00	Bacteroidota	Bacteroidia	*Muribaculaceae*	*Vibrio*	*Vibrio parahaemolyticus*
Zotu6700	0.01	0.00	Bacteroidota	Bacteroidia	*Muribaculaceae*	*Vibrio*	*Vibrio parahaemolyticus*
Zotu5502	0.06	0.00	Firmicutes A	Clostridia	*Lachnospiraceae*	*1XD8-76*	*1XD8-76 sp003611955*
Zotu4372	0.05	0.00	Firmicutes A	Clostridia	*Lachnospiraceae*	*1XD8-76*	*1XD8-76 sp003611955*
Zotu5712	0.55	0.01	Firmicutes A	Clostridia	*Lachnospiraceae*	*Roseburia*	*Roseburia intestinalis*
Zotu4829	0.03	0.00	Firmicutes A	Clostridia	*UBA1381*	*CAG-41*	*CAG-41 sp900066215*
Zotu3815	0.09	0.00	Firmicutes A	Clostridia	*UBA1381*	*CAG-41*	*CAG-41 sp900066215*
Zotu6673	0.02	0.00	Firmicutes A	Clostridia	*UBA1381*	*CAG-41*	*CAG-41 sp900066215*
Zotu5719	0.02	0.00	Firmicutes A	Clostridia	*UBA1381*	*CAG-41*	*CAG-41 sp900066215*
Zotu6477	0.03	0.00	Firmicutes A	Clostridia	*UBA1381*	*CAG-41*	*CAG-41 sp900066215*
Zotu4238	0.08	0.00	Firmicutes A	Clostridia	*UBA1381*	*CAG-41*	*CAG-41 sp900066215*
Zotu4536	0.09	0.00	Firmicutes A	Clostridia	*UBA1381*	*CAG-41*	*CAG-41 sp900066215*
Zotu4850	0.05	0.00	Firmicutes A	Clostridia	*UBA1381*	*CAG-41*	*CAG-41 sp900066215*
Zotu4676	0.03	0.00	Firmicutes A	Clostridia	*UBA1381*	*CAG-41*	*CAG-41 sp900066215*

Zotu4177	0.07	0.00	Firmicutes A	Clostridia	*UBA1381*	*CAG-41*	*CAG-41 sp900066215*
Zotu5450	0.01	0.00	Firmicutes A	Clostridia	*Oscillospiraceae*	*CAG-83*	*CAG-83 sp000431575*
Zotu6368	0.40	0.01	Proteobacteria	Gammaproteobacteria	*Enterobacteriaceae*	*Cronobacter*	*Cronobacter sakazakii*
Zotu439	0.03	0.00	Proteobacteria	Gammaproteobacteria	*Enterobacteriaceae*	*Salmonella*	*Salmonella enterica*
Zotu693	0.07	0.01	Proteobacteria	Gammaproteobacteria	*Enterobacteriaceae*	*Salmonella*	*Salmonella enterica*
Zotu2590	0.01	0.00	Proteobacteria	Gammaproteobacteria	*Enterobacteriaceae*	*Salmonella*	*Salmonella enterica*
Zotu1178	0.02	0.00	Proteobacteria	Gammaproteobacteria	*Enterobacteriaceae*	*Salmonella*	*Salmonella enterica*
Zotu503	0.02	0.00	Proteobacteria	Gammaproteobacteria	*Enterobacteriaceae*	*Salmonella*	*Salmonella enterica*
Zotu1538	0.01	0.00	Proteobacteria	Gammaproteobacteria	*Enterobacteriaceae*	*Salmonella*	*Salmonella enterica*
Zotu521	0.04	0.00	Proteobacteria	Gammaproteobacteria	*Enterobacteriaceae*	*Salmonella*	*Salmonella enterica*
Zotu800	0.03	0.00	Proteobacteria	Gammaproteobacteria	*Enterobacteriaceae*	*Salmonella*	*Salmonella enterica*
Zotu446	0.03	0.00	Proteobacteria	Gammaproteobacteria	*Enterobacteriaceae*	*Salmonella*	*Salmonella enterica*
Zotu438	0.02	0.00	Proteobacteria	Gammaproteobacteria	*Enterobacteriaceae*	*Salmonella*	*Salmonella enterica*
Zotu581	0.03	0.00	Proteobacteria	Gammaproteobacteria	*Enterobacteriaceae*	*Salmonella*	*Salmonella enterica*
Zotu3454	0.03	0.00	Proteobacteria	Gammaproteobacteria	*Vibrionaceae*	*Vibrio*	*Vibrio parahaemolyticus*
Zotu5267	0.02	0.00	Proteobacteria	Gammaproteobacteria	*Vibrionaceae*	*Vibrio*	*Vibrio parahaemolyticus*
Zotu3400	0.03	0.00	Proteobacteria	Gammaproteobacteria	*Vibrionaceae*	*Vibrio*	*Vibrio parahaemolyticus*
Zotu2297	0.04	0.00	Proteobacteria	Gammaproteobacteria	*Vibrionaceae*	*Vibrio*	*Vibrio parahaemolyticus*
Zotu3049	0.05	0.00	Proteobacteria	Gammaproteobacteria	*Vibrionaceae*	*Vibrio*	*Vibrio parahaemolyticus*
Zotu6568	0.02	0.00	Proteobacteria	Gammaproteobacteria	*Vibrionaceae*	*Vibrio*	*Vibrio parahaemolyticus*
Zotu3272	0.03	0.00	Proteobacteria	Gammaproteobacteria	*Vibrionaceae*	*Vibrio*	*Vibrio parahaemolyticus*
Zotu3646	0.01	0.00	Proteobacteria	Gammaproteobacteria	*Vibrionaceae*	*Vibrio*	*Vibrio parahaemolyticus*
Zotu6219	0.04	0.00	Proteobacteria	Gammaproteobacteria	*Vibrionaceae*	*Vibrio*	*Vibrio parahaemolyticus*
Zotu5169	0.25	0.01	Proteobacteria	Gammaproteobacteria	*Vibrionaceae*	*Vibrio*	*Vibrio parahaemolyticus*
Zotu3392	0.02	0.00	Proteobacteria	Gammaproteobacteria	*Vibrionaceae*	*Vibrio*	*Vibrio parahaemolyticus*
Zotu3803	0.01	0.00	Proteobacteria	Gammaproteobacteria	*Vibrionaceae*	*Vibrio*	*Vibrio parahaemolyticus*
Zotu2412	0.05	0.00	Proteobacteria	Gammaproteobacteria	*Vibrionaceae*	*Vibrio*	*Vibrio parahaemolyticus*
Zotu2947	0.02	0.00	Proteobacteria	Gammaproteobacteria	*Vibrionaceae*	*Vibrio*	*Vibrio parahaemolyticus*
Zotu3687	0.02	0.00	Proteobacteria	Gammaproteobacteria	*Vibrionaceae*	*Vibrio*	*Vibrio parahaemolyticus*
Zotu2594	0.06	0.00	Proteobacteria	Gammaproteobacteria	*Vibrionaceae*	*Vibrio*	*Vibrio parahaemolyticus*
Zotu2835	0.05	0.00	Proteobacteria	Gammaproteobacteria	*Vibrionaceae*	*Vibrio*	*Vibrio parahaemolyticus*
Zotu4806	0.02	0.00	Proteobacteria	Gammaproteobacteria	*Vibrionaceae*	*Vibrio*	*Vibrio parahaemolyticus*
Zotu2420	0.02	0.00	Proteobacteria	Gammaproteobacteria	*Vibrionaceae*	*Vibrio*	*Vibrio parahaemolyticus*
Zotu4966	0.05	0.00	Proteobacteria	Gammaproteobacteria	*Vibrionaceae*	*Vibrio*	*Vibrio parahaemolyticus*
Zotu2666	0.01	0.00	Proteobacteria	Gammaproteobacteria	*Vibrionaceae*	*Vibrio*	*Vibrio parahaemolyticus*
Zotu3351	0.01	0.00	Proteobacteria	Gammaproteobacteria	*Vibrionaceae*	*Vibrio*	*Vibrio parahaemolyticus*
Zotu2854	0.03	0.00	Proteobacteria	Gammaproteobacteria	*Vibrionaceae*	*Vibrio*	*Vibrio parahaemolyticus*
Zotu3370	0.02	0.00	Proteobacteria	Gammaproteobacteria	*Vibrionaceae*	*Vibrio*	*Vibrio parahaemolyticus*
Zotu4366	0.04	0.00	Proteobacteria	Gammaproteobacteria	*Vibrionaceae*	*Vibrio*	*Vibrio parahaemolyticus*
Zotu3675	0.05	0.00	Proteobacteria	Gammaproteobacteria	*Vibrionaceae*	*Vibrio*	*Vibrio parahaemolyticus*
Zotu1922	0.02	0.00	Proteobacteria	Gammaproteobacteria	*Vibrionaceae*	*Vibrio*	*Vibrio parahaemolyticus*

Zotu2485	0.03	0.00	Proteobacteria	Gammaproteobacteria	*Vibrionaceae*	*Vibrio*	*Vibrio parahaemolyticus*
Zotu3258	0.02	0.00	Proteobacteria	Gammaproteobacteria	*Vibrionaceae*	*Vibrio*	*Vibrio parahaemolyticus*
Zotu6208	0.02	0.00	Proteobacteria	Gammaproteobacteria	*Vibrionaceae*	*Vibrio*	*Vibrio parahaemolyticus*
Zotu4343	0.03	0.00	Proteobacteria	Gammaproteobacteria	*Vibrionaceae*	*Vibrio*	*Vibrio parahaemolyticus*
Zotu2113	0.02	0.00	Proteobacteria	Gammaproteobacteria	*Vibrionaceae*	*Vibrio*	*Vibrio parahaemolyticus*
Zotu2298	0.11	0.00	Proteobacteria	Gammaproteobacteria	*Vibrionaceae*	*Vibrio*	*Vibrio parahaemolyticus*
Zotu3607	0.06	0.00	Proteobacteria	Gammaproteobacteria	*Vibrionaceae*	*Vibrio*	*Vibrio parahaemolyticus*
Zotu3136	0.04	0.00	Proteobacteria	Gammaproteobacteria	*Vibrionaceae*	*Vibrio*	*Vibrio parahaemolyticus*
Zotu2884	0.02	0.00	Proteobacteria	Gammaproteobacteria	*Vibrionaceae*	*Vibrio*	*Vibrio parahaemolyticus*
Zotu4031	0.06	0.00	Proteobacteria	Gammaproteobacteria	*Vibrionaceae*	*Vibrio*	*Vibrio parahaemolyticus*
Zotu3846	0.03	0.00	Proteobacteria	Gammaproteobacteria	*Vibrionaceae*	*Vibrio*	*Vibrio parahaemolyticus*
Zotu4222	0.04	0.00	Proteobacteria	Gammaproteobacteria	*Vibrionaceae*	*Vibrio*	*Vibrio parahaemolyticus*
Zotu3331	0.02	0.00	Proteobacteria	Gammaproteobacteria	*Vibrionaceae*	*Vibrio*	*Vibrio parahaemolyticus*
Zotu3760	0.02	0.00	Proteobacteria	Gammaproteobacteria	*Vibrionaceae*	*Vibrio*	*Vibrio parahaemolyticus*
Zotu4200	0.02	0.00	Proteobacteria	Gammaproteobacteria	*Vibrionaceae*	*Vibrio*	*Vibrio parahaemolyticus*
Zotu5557	0.01	0.00	Proteobacteria	Gammaproteobacteria	*Vibrionaceae*	*Vibrio*	*Vibrio parahaemolyticus*
Zotu2963	0.02	0.00	Proteobacteria	Gammaproteobacteria	*Vibrionaceae*	*Vibrio*	*Vibrio parahaemolyticus*
Zotu3301	0.01	0.00	Proteobacteria	Gammaproteobacteria	*Vibrionaceae*	*Vibrio*	*Vibrio parahaemolyticus*
Zotu3074	0.02	0.00	Proteobacteria	Gammaproteobacteria	*Vibrionaceae*	*Vibrio*	*Vibrio parahaemolyticus*
Zotu5544	0.01	0.00	Proteobacteria	Gammaproteobacteria	*Vibrionaceae*	*Vibrio*	*Vibrio parahaemolyticus*
Zotu5883	0.02	0.00	Proteobacteria	Gammaproteobacteria	*Vibrionaceae*	*Vibrio*	*Vibrio parahaemolyticus*
Zotu2292	0.02	0.00	Proteobacteria	Gammaproteobacteria	*Vibrionaceae*	*Vibrio*	*Vibrio parahaemolyticus*
Zotu2230	0.02	0.00	Proteobacteria	Gammaproteobacteria	*Vibrionaceae*	*Vibrio*	*Vibrio parahaemolyticus*
Zotu4581	0.01	0.00	Proteobacteria	Gammaproteobacteria	*Vibrionaceae*	*Vibrio*	*Vibrio parahaemolyticus*
Zotu2067	0.08	0.00	Proteobacteria	Gammaproteobacteria	*Vibrionaceae*	*Vibrio*	*Vibrio parahaemolyticus*
Zotu3093	0.01	0.00	Proteobacteria	Gammaproteobacteria	*Vibrionaceae*	*Vibrio*	*Vibrio parahaemolyticus*
Zotu3468	0.05	0.00	Proteobacteria	Gammaproteobacteria	*Vibrionaceae*	*Vibrio*	*Vibrio parahaemolyticus*
Zotu4382	0.01	0.00	Proteobacteria	Gammaproteobacteria	*Vibrionaceae*	*Vibrio*	*Vibrio parahaemolyticus*
Zotu4807	0.01	0.00	Proteobacteria	Gammaproteobacteria	*Vibrionaceae*	*Vibrio*	*Vibrio parahaemolyticus*
Zotu6633	0.02	0.00	Proteobacteria	Gammaproteobacteria	*Vibrionaceae*	*Vibrio*	*Vibrio parahaemolyticus*
Zotu3573	0.02	0.00	Proteobacteria	Gammaproteobacteria	*Vibrionaceae*	*Vibrio*	*Vibrio parahaemolyticus*
Zotu2691	0.08	0.00	Proteobacteria	Gammaproteobacteria	*Vibrionaceae*	*Vibrio*	*Vibrio parahaemolyticus*
Zotu2164	0.02	0.00	Proteobacteria	Gammaproteobacteria	*Vibrionaceae*	*Vibrio*	*Vibrio parahaemolyticus*
Zotu3751	0.03	0.00	Proteobacteria	Gammaproteobacteria	*Vibrionaceae*	*Vibrio*	*Vibrio parahaemolyticus*
Zotu4188	0.02	0.00	Proteobacteria	Gammaproteobacteria	*Vibrionaceae*	*Vibrio*	*Vibrio parahaemolyticus*
Zotu4763	0.03	0.00	Proteobacteria	Gammaproteobacteria	*Vibrionaceae*	*Vibrio*	*Vibrio parahaemolyticus*
Zotu2610	0.07	0.00	Proteobacteria	Gammaproteobacteria	*Vibrionaceae*	*Vibrio*	*Vibrio parahaemolyticus*
Zotu4035	0.02	0.00	Proteobacteria	Gammaproteobacteria	*Vibrionaceae*	*Vibrio*	*Vibrio parahaemolyticus*
Zotu5273	0.01	0.00	Proteobacteria	Gammaproteobacteria	*Vibrionaceae*	*Vibrio*	*Vibrio parahaemolyticus*

Table S4. Differential zOTU abundance between H and HCP rats.

zOTU_ID	H%	HCP%	Phylum	Class	Family	Genus	Species
Zotu5206	0.00	0.08	Actinobacteriota	Coriobacteriia	*Eggerthellaceae*	*CAG-1427*	*CAG-1427 sp000435675*
Zotu5890	0.03	0.00	Bacteroidota	Bacteroidia	*Muribaculaceae*	*Muribaculum*	*Muribaculum sp001701195*
Zotu5281	0.08	0.00	Bacteroidota	Bacteroidia	*Muribaculaceae*	*Muribaculum*	*Muribaculum sp001701195*
Zotu4405	0.05	0.00	Bacteroidota	Bacteroidia	*Muribaculaceae*	*Muribaculum*	*Muribaculum sp001701195*
Zotu5561	0.11	0.00	Bacteroidota	Bacteroidia	*Muribaculaceae*	*Muribaculum*	*Muribaculum sp002492595*
Zotu4473	0.14	0.00	Bacteroidota	Bacteroidia	*Muribaculaceae*	*Muribaculum*	*Muribaculum sp002492595*
Zotu5048	0.06	0.00	Bacteroidota	Bacteroidia	*Muribaculaceae*	*Muribaculum*	*Muribaculum sp002492595*
Zotu4025	0.04	0.00	Bacteroidota	Bacteroidia	*Muribaculaceae*	*Muribaculum*	*Muribaculum sp002492595*
Zotu5809	0.12	0.00	Bacteroidota	Bacteroidia	*Muribaculaceae*	*Muribaculum*	*Muribaculum sp002492595*
Zotu4089	0.17	0.00	Bacteroidota	Bacteroidia	*Muribaculaceae*	*Muribaculum*	*Muribaculum sp002492595*
Zotu4227	0.09	0.00	Bacteroidota	Bacteroidia	*Muribaculaceae*	*Muribaculum*	*Muribaculum sp002492595*
Zotu5217	0.06	0.00	Bacteroidota	Bacteroidia	*Muribaculaceae*	*Muribaculum*	*Muribaculum sp002492595*
Zotu5577	0.05	0.00	Bacteroidota	Bacteroidia	*Muribaculaceae*	*Muribaculum*	*Muribaculum sp002492595*
Zotu4197	0.18	0.00	Bacteroidota	Bacteroidia	*Muribaculaceae*	*Muribaculum*	*Muribaculum sp002492595*
Zotu2681	0.11	0.00	Bacteroidota	Bacteroidia	*Muribaculaceae*	*Muribaculum*	*Muribaculum sp002492595*
Zotu3028	0.06	0.00	Bacteroidota	Bacteroidia	*Muribaculaceae*	*Muribaculum*	*Muribaculum sp002492595*
Zotu4991	0.04	0.00	Bacteroidota	Bacteroidia	*Muribaculaceae*	*Muribaculum*	*Muribaculum sp002492595*
Zotu6229	0.02	0.00	Bacteroidota	Bacteroidia	*Muribaculaceae*	*Muribaculum*	*Muribaculum sp002492595*
Zotu6469	0.03	0.00	Bacteroidota	Bacteroidia	*Muribaculaceae*	*Muribaculum*	*Muribaculum sp002492595*
Zotu6156	0.10	0.00	Bacteroidota	Bacteroidia	*Muribaculaceae*	*Muribaculum*	*Muribaculum sp002492595*
Zotu2423	0.04	0.00	Bacteroidota	Bacteroidia	*Muribaculaceae*	*Muribaculum*	*Muribaculum sp002492595*
Zotu4819	0.07	0.00	Bacteroidota	Bacteroidia	*Muribaculaceae*	*Muribaculum*	*Muribaculum sp002492595*
Zotu5215	0.04	0.00	Bacteroidota	Bacteroidia	*Muribaculaceae*	*Muribaculum*	*Muribaculum sp002492595*
Zotu2528	0.04	0.00	Bacteroidota	Bacteroidia	*Muribaculaceae*	*Muribaculum*	*Muribaculum sp002492595*
Zotu5445	0.02	0.00	Bacteroidota	Bacteroidia	*Muribaculaceae*	*Muribaculum*	*Muribaculum sp002492595*
Zotu5255	0.08	0.00	Bacteroidota	Bacteroidia	*Muribaculaceae*	*Muribaculum*	*Muribaculum sp002492595*
Zotu5862	0.03	0.00	Bacteroidota	Bacteroidia	*Muribaculaceae*	*Muribaculum*	*Muribaculum sp002492595*
Zotu4527	0.06	0.00	Bacteroidota	Bacteroidia	*Muribaculaceae*	*Muribaculum*	*Muribaculum sp002492595*
Zotu6607	0.07	0.00	Bacteroidota	Bacteroidia	*Muribaculaceae*	*Muribaculum*	*Muribaculum sp002492595*
Zotu3584	0.03	0.00	Bacteroidota	Bacteroidia	*Muribaculaceae*	*Muribaculum*	*Muribaculum sp002492595*
Zotu6093	0.03	0.00	Bacteroidota	Bacteroidia	*Muribaculaceae*	*Muribaculum*	*Muribaculum sp002492595*
Zotu6638	0.07	0.00	Bacteroidota	Bacteroidia	*Muribaculaceae*	*Muribaculum*	*Muribaculum sp002492595*

Zotu4859	0.04	0.00	Bacteroidota	Bacteroidia	*Muribaculaceae*	*Muribaculum*	*Muribaculum sp002492595*
Zotu5152	0.03	0.00	Bacteroidota	Bacteroidia	*Muribaculaceae*	*Muribaculum*	*Muribaculum sp002492595*
Zotu6493	0.02	0.00	Bacteroidota	Bacteroidia	*Muribaculaceae*	*Muribaculum*	*Muribaculum sp002492595*
Zotu6097	0.03	0.00	Bacteroidota	Bacteroidia	*Muribaculaceae*	*Muribaculum*	*Muribaculum sp002492595*
Zotu5484	0.04	0.00	Bacteroidota	Bacteroidia	*Muribaculaceae*	*Muribaculum*	*Muribaculum sp002492595*
Zotu5534	0.02	0.00	Bacteroidota	Bacteroidia	*Muribaculaceae*	*Muribaculum*	*Muribaculum sp002492595*
Zotu6489	0.03	0.00	Bacteroidota	Bacteroidia	*Muribaculaceae*	*Muribaculum*	*Muribaculum sp002492595*
Zotu6460	0.02	0.00	Bacteroidota	Bacteroidia	*Muribaculaceae*	*Muribaculum*	*Muribaculum sp002492595*
Zotu5572	0.02	0.00	Bacteroidota	Bacteroidia	*Muribaculaceae*	*Muribaculum*	*Muribaculum sp002492595*
Zotu6595	0.04	0.00	Bacteroidota	Bacteroidia	*Muribaculaceae*	*Muribaculum*	*Muribaculum sp002492595*
Zotu5658	0.02	0.00	Bacteroidota	Bacteroidia	*Muribaculaceae*	*Muribaculum*	*Muribaculum sp002492595*
Zotu5634	0.02	0.00	Bacteroidota	Bacteroidia	*Muribaculaceae*	*Muribaculum*	*Muribaculum sp002492595*
Zotu5807	0.03	0.00	Bacteroidota	Bacteroidia	*Muribaculaceae*	*Muribaculum*	*Muribaculum sp002492595*
Zotu5716	0.02	0.00	Bacteroidota	Bacteroidia	*Muribaculaceae*	*Muribaculum*	*Muribaculum sp002492595*
Zotu5951	0.02	0.00	Bacteroidota	Bacteroidia	*Muribaculaceae*	*Muribaculum*	*Muribaculum sp002492595*
Zotu3894	0.04	0.00	Bacteroidota	Bacteroidia	*Muribaculaceae*	*Muribaculum*	*Muribaculum sp002492595*
Zotu5743	0.02	0.00	Bacteroidota	Bacteroidia	*Muribaculaceae*	*Muribaculum*	*Muribaculum sp002492595*
Zotu4941	0.07	0.00	Bacteroidota	Bacteroidia	*Muribaculaceae*	*Muribaculum*	*Vibrio parahaemolyticus*
Zotu5804	0.04	0.00	Bacteroidota	Bacteroidia	*Muribaculaceae*	*UBA7173*	*UBA7173 sp004102805*
Zotu4753	0.05	0.00	Bacteroidota	Bacteroidia	*Muribaculaceae*	*UBA7173*	*UBA7173 sp004102805*
Zotu6105	0.05	0.00	Bacteroidota	Bacteroidia	*Muribaculaceae*	*Vibrio*	*Vibrio parahaemolyticus*
Zotu6700	0.03	0.00	Bacteroidota	Bacteroidia	*Muribaculaceae*	*Vibrio*	*Vibrio parahaemolyticus*
Zotu4475	0.05	0.00	Bacteroidota	Bacteroidia	*Muribaculaceae*	*Vibrio*	*Vibrio parahaemolyticus*
Zotu5865	0.03	0.00	Desulfobacterota	Desulfovibrionia	*Desulfovibrionaceae*	*Desulfovibrio*	*Desulfovibrio sp003860215*
Zotu3742	0.06	0.00	Desulfobacterota	Desulfovibrionia	*Desulfovibrionaceae*	*Desulfovibrio*	*Desulfovibrio sp003860215*
Zotu5370	0.02	0.00	Desulfobacterota	Desulfovibrionia	*Desulfovibrionaceae*	*Desulfovibrio*	*Desulfovibrio sp003860215*
Zotu3774	0.02	0.00	Desulfobacterota	Desulfovibrionia	*Desulfovibrionaceae*	*Desulfovibrio*	*Desulfovibrio sp003860215*
Zotu3238	0.02	0.00	Desulfobacterota	Desulfovibrionia	*Desulfovibrionaceae*	*Desulfovibrio*	*Desulfovibrio sp900547595*
Zotu4155	0.05	0.00	Desulfobacterota	Desulfovibrionia	*Desulfovibrionaceae*	*Desulfovibrio*	*Desulfovibrio sp900547595*
Zotu5342	0.01	0.00	Desulfobacterota	Desulfovibrionia	*Desulfovibrionaceae*	*Desulfovibrio*	*Desulfovibrio sp900547595*
Zotu3812	0.04	0.00	Desulfobacterota	Desulfovibrionia	*Desulfovibrionaceae*	*Desulfovibrio*	*Desulfovibrio sp900547595*
Zotu4998	0.02	0.00	Desulfobacterota	Desulfovibrionia	*Desulfovibrionaceae*	*Desulfovibrio*	*Desulfovibrio sp900547595*
Zotu4009	0.03	0.00	Desulfobacterota	Desulfovibrionia	*Desulfovibrionaceae*	*Desulfovibrio*	*Desulfovibrio sp900547595*
Zotu6629	0.02	0.00	Desulfobacterota	Desulfovibrionia	*Desulfovibrionaceae*	*Desulfovibrio*	*Desulfovibrio sp900547595*

Zotu4327	0.02	0.00	Desulfobacterota	Desulfovibrionia	*Desulfovibrionaceae*	*Desulfovibrio*	*Desulfovibrio sp900547595*
Zotu4782	0.03	0.00	Desulfobacterota	Desulfovibrionia	*Desulfovibrionaceae*	*Desulfovibrio*	*Desulfovibrio sp900547595*
Zotu4439	0.05	0.00	Desulfobacterota	Desulfovibrionia	*Desulfovibrionaceae*	*Desulfovibrio*	*Desulfovibrio sp900547595*
Zotu6265	0.02	0.00	Desulfobacterota	Desulfovibrionia	*Desulfovibrionaceae*	*Desulfovibrio*	*Desulfovibrio sp900547595*
Zotu4964	0.01	0.00	Desulfobacterota	Desulfovibrionia	*Desulfovibrionaceae*	*Desulfovibrio*	*Desulfovibrio sp900547595*
Zotu5179	0.00	0.03	Firmicutes A	Clostridia	*Clostridiaceae*	*Clostridium*	*Clostridium saudiense*
Zotu6695	0.00	0.21	Firmicutes A	Clostridia	*Lachnospiraceae*	*Bacteroides F*	*Bacteroides_F pectinophilus*
Zotu5489	0.00	0.46	Firmicutes A	Clostridia	*Lachnospiraceae*	*Bacteroides F*	*Bacteroides_F pectinophilus*
Zotu5932	0.00	0.14	Firmicutes A	Clostridia	*Lachnospiraceae*	*Bacteroides F*	*Bacteroides_F pectinophilus*
Zotu6424	0.00	0.23	Firmicutes A	Clostridia	*Lachnospiraceae*	*Bacteroides F*	*Bacteroides_F pectinophilus*
Zotu4341	0.00	0.29	Firmicutes A	Clostridia	*Lachnospiraceae*	*Bacteroides F*	*Bacteroides_F pectinophilus*
Zotu6321	0.80	0.00	Firmicutes A	Clostridia	*Lachnospiraceae*	*Enterocloster*	*Acetatifactor sp900066565*
Zotu6659	0.01	0.00	Firmicutes A	Clostridia	*Lachnospiraceae*	*Kineothrix*	*Kineothrix alysoides*
Zotu1092	0.00	0.04	Proteobacteria	Gammaproteobacteria	*Enterobacteriaceae*	*Cronobacter*	*Akkermansia muciniphila*
Zotu1361	0.00	0.02	Proteobacteria	Gammaproteobacteria	*Enterobacteriaceae*	*Cronobacter*	*Akkermansia muciniphila*
Zotu682	0.00	0.03	Proteobacteria	Gammaproteobacteria	*Enterobacteriaceae*	*Cronobacter*	*Akkermansia muciniphila*
Zotu627	0.00	0.03	Proteobacteria	Gammaproteobacteria	*Enterobacteriaceae*	*Cronobacter*	*Cronobacter malonaticus*
Zotu832	0.00	0.02	Proteobacteria	Gammaproteobacteria	*Enterobacteriaceae*	*Cronobacter*	*Cronobacter malonaticus*
Zotu861	0.00	0.01	Proteobacteria	Gammaproteobacteria	*Enterobacteriaceae*	*Cronobacter*	*Cronobacter malonaticus*
Zotu773	0.00	0.01	Proteobacteria	Gammaproteobacteria	*Enterobacteriaceae*	*Cronobacter*	*Cronobacter malonaticus*
Zotu1665	0.00	0.02	Proteobacteria	Gammaproteobacteria	*Enterobacteriaceae*	*Cronobacter*	*Cronobacter malonaticus*
Zotu595	0.00	0.08	Proteobacteria	Gammaproteobacteria	*Enterobacteriaceae*	*Cronobacter*	*Cronobacter malonaticus*
Zotu1458	0.00	0.01	Proteobacteria	Gammaproteobacteria	*Enterobacteriaceae*	*Cronobacter*	*Cronobacter malonaticus*
Zotu445	0.00	0.09	Proteobacteria	Gammaproteobacteria	*Enterobacteriaceae*	*Cronobacter*	*Cronobacter malonaticus*
Zotu352	0.00	0.06	Proteobacteria	Gammaproteobacteria	*Enterobacteriaceae*	*Cronobacter*	*Cronobacter malonaticus*
Zotu1176	0.00	0.01	Proteobacteria	Gammaproteobacteria	*Enterobacteriaceae*	*Cronobacter*	*Cronobacter malonaticus*
Zotu1279	0.00	0.04	Proteobacteria	Gammaproteobacteria	*Enterobacteriaceae*	*Cronobacter*	*Cronobacter malonaticus*
Zotu827	0.00	0.02	Proteobacteria	Gammaproteobacteria	*Enterobacteriaceae*	*Cronobacter*	*Cronobacter malonaticus*
Zotu610	0.00	0.03	Proteobacteria	Gammaproteobacteria	*Enterobacteriaceae*	*Cronobacter*	*Cronobacter malonaticus*
Zotu818	0.00	0.02	Proteobacteria	Gammaproteobacteria	*Enterobacteriaceae*	*Cronobacter*	*Cronobacter malonaticus*
Zotu931	0.00	0.02	Proteobacteria	Gammaproteobacteria	*Enterobacteriaceae*	*Cronobacter*	*Cronobacter malonaticus*
Zotu1470	0.00	0.01	Proteobacteria	Gammaproteobacteria	*Enterobacteriaceae*	*Cronobacter*	*Cronobacter malonaticus*
Zotu1322	0.00	0.01	Proteobacteria	Gammaproteobacteria	*Enterobacteriaceae*	*Cronobacter*	*Cronobacter malonaticus*
Zotu1822	0.00	0.01	Proteobacteria	Gammaproteobacteria	*Enterobacteriaceae*	*Cronobacter*	*Cronobacter malonaticus*

Zotu406	0.00	0.03	Proteobacteria	Gammaproteobacteria	*Enterobacteriaceae*	*Cronobacter*	*Cronobacter malonaticus*
Zotu855	0.00	0.03	Proteobacteria	Gammaproteobacteria	*Enterobacteriaceae*	*Cronobacter*	*Cronobacter sakazakii*
Zotu1009	0.00	0.02	Proteobacteria	Gammaproteobacteria	*Enterobacteriaceae*	*Cronobacter*	*Cronobacter sakazakii*
Zotu918	0.00	0.03	Proteobacteria	Gammaproteobacteria	*Enterobacteriaceae*	*Cronobacter*	*Cronobacter sakazakii*
Zotu428	0.00	0.02	Proteobacteria	Gammaproteobacteria	*Enterobacteriaceae*	*Cronobacter*	*Cronobacter sakazakii*
Zotu1658	0.00	0.03	Proteobacteria	Gammaproteobacteria	*Enterobacteriaceae*	*Cronobacter*	*Cronobacter sakazakii*
Zotu765	0.00	0.02	Proteobacteria	Gammaproteobacteria	*Enterobacteriaceae*	*Cronobacter*	*Cronobacter sakazakii*
Zotu888	0.00	0.01	Proteobacteria	Gammaproteobacteria	*Enterobacteriaceae*	*Cronobacter*	*Cronobacter sakazakii*
Zotu1081	0.00	0.01	Proteobacteria	Gammaproteobacteria	*Enterobacteriaceae*	*Cronobacter*	*Cronobacter sakazakii*
Zotu781	0.00	0.01	Proteobacteria	Gammaproteobacteria	*Enterobacteriaceae*	*Cronobacter*	*Cronobacter sakazakii*
Zotu720	0.00	0.07	Proteobacteria	Gammaproteobacteria	*Enterobacteriaceae*	*Cronobacter*	*Cronobacter sakazakii*
Zotu878	0.00	0.06	Proteobacteria	Gammaproteobacteria	*Enterobacteriaceae*	*Cronobacter*	*Cronobacter sakazakii*
Zotu1000	0.00	0.01	Proteobacteria	Gammaproteobacteria	*Enterobacteriaceae*	*Cronobacter*	*Cronobacter sakazakii*
Zotu1316	0.00	0.01	Proteobacteria	Gammaproteobacteria	*Enterobacteriaceae*	*Cronobacter*	*Cronobacter sakazakii*
Zotu1523	0.00	0.01	Proteobacteria	Gammaproteobacteria	*Enterobacteriaceae*	*Cronobacter*	*Cronobacter sakazakii*
Zotu706	0.00	0.03	Proteobacteria	Gammaproteobacteria	*Enterobacteriaceae*	*Cronobacter*	*Cronobacter sakazakii*
Zotu1909	0.00	0.01	Proteobacteria	Gammaproteobacteria	*Enterobacteriaceae*	*Cronobacter*	*Cronobacter sakazakii*
Zotu1152	0.00	0.02	Proteobacteria	Gammaproteobacteria	*Enterobacteriaceae*	*Cronobacter*	*Cronobacter sakazakii*
Zotu1367	0.00	0.02	Proteobacteria	Gammaproteobacteria	*Enterobacteriaceae*	*Cronobacter*	*Cronobacter sakazakii*
Zotu1720	0.00	0.01	Proteobacteria	Gammaproteobacteria	*Enterobacteriaceae*	*Cronobacter*	*Cronobacter sakazakii*
Zotu381	0.00	0.05	Proteobacteria	Gammaproteobacteria	*Enterobacteriaceae*	*Cronobacter*	*Cronobacter sakazakii*
Zotu736	0.00	0.04	Proteobacteria	Gammaproteobacteria	*Enterobacteriaceae*	*Cronobacter*	*Cronobacter sakazakii*
Zotu1077	0.00	0.04	Proteobacteria	Gammaproteobacteria	*Enterobacteriaceae*	*Cronobacter*	*Cronobacter sakazakii*
Zotu997	0.00	0.01	Proteobacteria	Gammaproteobacteria	*Enterobacteriaceae*	*Cronobacter*	*Cronobacter sakazakii*
Zotu974	0.00	0.02	Proteobacteria	Gammaproteobacteria	*Enterobacteriaceae*	*Cronobacter*	*Cronobacter sakazakii*
Zotu1038	0.00	0.02	Proteobacteria	Gammaproteobacteria	*Enterobacteriaceae*	*Cronobacter*	*Cronobacter sakazakii*
Zotu1432	0.00	0.02	Proteobacteria	Gammaproteobacteria	*Enterobacteriaceae*	*Cronobacter*	*Cronobacter sakazakii*
Zotu726	0.00	0.02	Proteobacteria	Gammaproteobacteria	*Enterobacteriaceae*	*Cronobacter*	*Cronobacter sakazakii*
Zotu1293	0.00	0.01	Proteobacteria	Gammaproteobacteria	*Enterobacteriaceae*	*Cronobacter*	*Cronobacter sakazakii*
Zotu1773	0.00	0.01	Proteobacteria	Gammaproteobacteria	*Enterobacteriaceae*	*Cronobacter*	*Cronobacter sakazakii*
Zotu576	0.00	0.03	Proteobacteria	Gammaproteobacteria	*Enterobacteriaceae*	*Cronobacter*	*Cronobacter sakazakii*
Zotu821	0.00	0.01	Proteobacteria	Gammaproteobacteria	*Enterobacteriaceae*	*Cronobacter*	*Cronobacter sakazakii*
Zotu695	0.00	0.15	Verrucomicrobiota	Verrucomicrobiae	*Akkermansiaceae*	*Akkermansia*	*Akkermansia muciniphila*
Zotu1477	0.00	0.02	Verrucomicrobiota	Verrucomicrobiae	*Akkermansiaceae*	*Akkermansia*	*Akkermansia muciniphila*

Zotu920	0.00	0.04	Verrucomicrobiota	Verrucomicrobiae	*Akkermansiaceae*	*Akkermansia*	*Akkermansia muciniphila*
Zotu505	0.00	0.04	Verrucomicrobiota	Verrucomicrobiae	*Akkermansiaceae*	*Akkermansia*	*Akkermansia muciniphila*
Zotu1011	0.00	0.05	Verrucomicrobiota	Verrucomicrobiae	*Akkermansiaceae*	*Akkermansia*	*Akkermansia muciniphila*
Zotu721	0.00	0.05	Verrucomicrobiota	Verrucomicrobiae	*Akkermansiaceae*	*Akkermansia*	*Akkermansia muciniphila*
Zotu724	0.00	0.04	Verrucomicrobiota	Verrucomicrobiae	*Akkermansiaceae*	*Akkermansia*	*Akkermansia muciniphila*
Zotu801	0.00	0.02	Verrucomicrobiota	Verrucomicrobiae	*Akkermansiaceae*	*Akkermansia*	*Akkermansia muciniphila*
Zotu1102	0.00	0.04	Verrucomicrobiota	Verrucomicrobiae	*Akkermansiaceae*	*Akkermansia*	*Akkermansia muciniphila*
Zotu1115	0.00	0.02	Verrucomicrobiota	Verrucomicrobiae	*Akkermansiaceae*	*Akkermansia*	*Akkermansia muciniphila*
Zotu1330	0.00	0.07	Verrucomicrobiota	Verrucomicrobiae	*Akkermansiaceae*	*Akkermansia*	*Akkermansia muciniphila*
Zotu1416	0.00	0.01	Verrucomicrobiota	Verrucomicrobiae	*Akkermansiaceae*	*Akkermansia*	*Akkermansia muciniphila*
Zotu1395	0.00	0.01	Verrucomicrobiota	Verrucomicrobiae	*Akkermansiaceae*	*Akkermansia*	*Akkermansia muciniphila*
Zotu1133	0.00	0.02	Verrucomicrobiota	Verrucomicrobiae	*Akkermansiaceae*	*Akkermansia*	*Akkermansia muciniphila*
Zotu1400	0.00	0.03	Verrucomicrobiota	Verrucomicrobiae	*Akkermansiaceae*	*Akkermansia*	*Akkermansia muciniphila*
Zotu740	0.00	0.04	Verrucomicrobiota	Verrucomicrobiae	*Akkermansiaceae*	*Akkermansia*	*Akkermansia muciniphila*
Zotu758	0.00	0.10	Verrucomicrobiota	Verrucomicrobiae	*Akkermansiaceae*	*Akkermansia*	*Akkermansia muciniphila*
Zotu1389	0.00	0.02	Verrucomicrobiota	Verrucomicrobiae	*Akkermansiaceae*	*Akkermansia*	*Akkermansia muciniphila*
Zotu1231	0.00	0.04	Verrucomicrobiota	Verrucomicrobiae	*Akkermansiaceae*	*Akkermansia*	*Akkermansia muciniphila*
Zotu1346	0.00	0.01	Verrucomicrobiota	Verrucomicrobiae	*Akkermansiaceae*	*Akkermansia*	*Akkermansia muciniphila*
Zotu757	0.00	0.04	Verrucomicrobiota	Verrucomicrobiae	*Akkermansiaceae*	*Akkermansia*	*Akkermansia muciniphila*
Zotu2262	0.00	0.01	Verrucomicrobiota	Verrucomicrobiae	*Akkermansiaceae*	*Akkermansia*	*Akkermansia muciniphila*
Zotu1160	0.00	0.01	Verrucomicrobiota	Verrucomicrobiae	*Akkermansiaceae*	*Akkermansia*	*Akkermansia muciniphila*
